# High-Energy Hybridized States Enable Long-Lived Hot Electrons in Cobaloxime-Silicon Nanocrystal System

**DOI:** 10.1021/jacs.5c19326

**Published:** 2026-02-09

**Authors:** Trung H. Le, Melissa K. Gish, Simran S. Saund, Taylor Aubry, Nathan R. Neale

**Affiliations:** † Materials, Chemical, and Computational Science Directorate, 53405 National Laboratory of the Rockies, Golden, Colorado 80401, United States; ‡ Renewable and Sustainable Energy Institute, University of Colorado Boulder, Boulder, Colorado 80309, United States

## Abstract

Strong electronic coupling is achieved between the molecular catalyst cobaloxime ([Co]) and silicon nanocrystals (Si NCs) bridged by an ethylenepyridine group derived from vinylpyridine (vpy) covalently bound to the Si NC surface (Si-vpy-[Co]). The ethylenepyridine tether in Si-vpy-[Co] is key to dramatic changes to the system’s physical properties—which are not observed in the corresponding formylpyridine (fpy) system (Si-fpy-[Co])—consistent with strong electronic coupling previously observed only in dark electrochemical systems. UV–vis absorption spectroscopy reveals new [Co]-centered electronic states in Si-vpy-[Co], and transient absorption spectroscopy finds a strong absorption feature appearing within 250 fs and persisting for at least 5 ns. Astoundingly, spectroelectrochemical measurements reveal that this absorption feature is consistent with both the singly reduced [Co]^−^ and doubly reduced [Co]^2–^ complexes, leading to the conclusion that these long-lived charges are derived from high-energy “hot” electrons residing in [Co]-centered states. Detailed analysis using cyclic voltammetry, spectroelectrochemistry, electron paramagnetic resonance spectroscopy, and density functional theory (DFT) calculations provides insight into the unique electronic structure created in Si-vpy-[Co]. DFT reveals that the new electronic states arise from hybridization between deep Si NC band states and high-energy molecular orbitals of the ethylenepyridine tether and the [Co] catalyst and are facilitated by σ-bonding character at the ethylenepyridine linkage. This study demonstrates that strong electronic coupling achieved through precise molecular chemistry can change the paradigm of otherwise fixed energy levels in hybrid photoelectrochemical systems for artificial photosynthesis and related applications.

## Graphical abstract

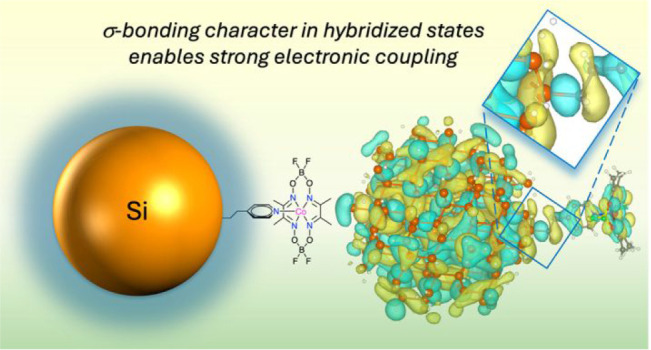

## Introduction

The success of photovoltaics, wind, and other variable energy resources^1^ has given commercial power providers many options for reliable, safe, and affordable electricity generation. These variable renewable energy sources can achieve grid penetration of up to 80% with diurnal storage (<12 h) provided by short-term reservoirs such as batteries.^2^ Methods for longer-duration storage include chemical fuels such as hydrogen and hydrocarbons that are potential complements to batteries by providing cost-effective energy storage to meet the world’s ever-increasing electricity demand.^3,4^ However, hydrogen produced from water electrolysis is unlikely to be cost-competitive compared with incumbent technologies such as pumped-hydro and compressed air energy storage for several decades unless significant capital cost improvements are achieved.^5,6^ Hydrocarbons such as methane produced as stored fuels also will face challenges competing with fossil energy sources relative to specialty chemicals that offer far more attractive near-term economics.^7^ Direct conversion of variable energy resources into stored chemical energy via artificial photosynthesis^8,9^ offers an alternative approach to multistep electricity generation and conversion^10^ as well as integrated photovoltaic and electrolysis approaches.^11^

The direct approach based on photoelectrochemical (PEC) conversion of sunlight into chemical bonds has been widely explored for over 50 years since the groundbreaking work of Fujishima and Honda, who achieved hydrogen production via PEC water splitting from a TiO_2_ semiconductor absorber,^12^ whereas in TiO_2_-catalyzed water oxidation, hydrogen was generated on a separate Pt electrode, showing the necessity of suitable catalysts for driving the overall water-splitting reaction. Heterogeneous catalysts have been integrated into PEC devices to improve catalytic performance for either the hydrogen evolution reaction (HER) or the carbon dioxide reduction reaction (CO_2_RR) with great success.^13–15^ Homogeneous molecular catalysts have also been “heterogenized” directly onto semiconductor surfaces by either chemisorption or physisorption and offer the ability to separately tune the light-harvesting and catalytic properties of the resulting hybrid PEC system.^16–18^

We have been exploring such molecular catalyst-semiconductor hybrid PEC systems by studying silicon nanocrystals (Si NCs) as models for bulk Si photocathodes to carefully understand the energetic requirements based on this technologically mature semiconductor. Our work has unveiled that the energetics of Lehn-type CO_2_RR molecular catalysts, such as Re­(bpy)­(CO)_3_Br (bpy = bipyridine), are generally energetically mismatched with bulk Si, and hybrid systems do not support unbiased photocatalysis as would be necessary in a 2-electrode PEC device.^19,20^ Potential solutions to overcoming this problem are to design a catalyst with a lower overpotential for the CO_2_RR or to select a less energetically demanding reaction such as HER.

A third potential approach is to generate an electronically coupled hybrid system where the molecular catalyst molecular orbitals hybridize with the semiconductor bands and provide new states that may lower the thermodynamic and kinetic barriers to photoinduced charge transfer (CT) and catalysis. Along these lines, recent work from Surendranath and coworkers has shown that a molecular redox couple conjugated within a graphitic carbon electrode results in inner-sphere CT from the electrode to the transition metal redox couple and allows its energy to float with the electrochemical potential applied to the system.^21–23^ Consequently, the classical outer-sphere CT process across the electrochemical double layer (EDL) is not observed, and cyclic voltammetry (CV) measurements show only EDL charging current and not redox features as would be expected in outer-sphere CT. Subsequent work by Surendranath and coworkers has found that CT to a heterogenized cobalt tetraphenylporphyrin (CoTPP) complex can be controlled by changing the electrolyte solvent from water to acetonitrile, where a flexible molecular tether allows the hydrophobic CoTPP catalyst to move into solution—outside the EDL—in acetonitrile, where outer-sphere charge transfer is observed.^24^ Conversely, in water, where the hydrophobic CoTPP complex prefers physisorption to the graphitic electrode, inner-sphere CT dominates. Recently, Hammes-Schiffer, Surendranath, and coworkers have found that electrochemically biasing the CoTPP-graphitic hybrid electrode in aqueous solution induces a concerted proton-coupled electron transfer (PCET) reaction to directly form a Co­(III) hydride via a “band-to-bond electron redistribution” where an electron from the graphitic electrode band states bypasses the Co­(II/I) redox couple and reacts with a proton from water to generate the Co­(III) hydride directly.^25^ This body of work inspired us to consider whether a similar strategy could be used to generate hybridized states in molecular catalyst-semiconductor systems.

Our recent work partially achieved this concept by directly tethering a Re-tricarbonyl bpy CO_2_RR electrocatalyst to a boron-doped Si NC via an sp^2^-hybridized carbon atom on the catalyst bpy ring, where this unique molecular tethering chemistry was achieved via a diazonium precursor [Re­(2,2′-bipyridyl-4-diazonium)­(CO)_3_Cl]­BF_4_.^20,26^ We used density functional theory (DFT) to show that hybridized states between the Si and Re electrocatalyst exist and experimentally confirmed that the surface-bound molecular catalyst perturbs the hybrid system’s electronic structure using transient absorption spectroscopy (TAS) and CV. However, our DFT calculations also showed that the hybridized states only form from the catalyst’s lowest unoccupied molecular orbital (LUMO). Importantly, the second reduction into the singly occupied molecular orbital (SOMO), which is necessary to consider for any 2e^–^ and 2H^+^ fuel-forming reaction such as CO_2_RR, requires significantly higher energy than that into the LUMO. This energetic arrangement prevents the second photoexcited electron necessary for CO_2_RR to be injected from the Si conduction band edge; thus, we found that photocatalysis proceeds at a rate similar to the Re catalyst alone with no Si present. We concluded that electrocatalysts with high redox potentials such as −2.2 V vs Fc^+/0^ needed for 2e^–^ reduction of Re­(bpy)­(CO)_3_Br are likely incompatible with Si semiconductor photocathodes for CO_2_RR without external bias or interfacial engineering that frequently are employed for Si photocathode-molecular CO_2_RR catalyst hybrid systems.^27–32^

In this work, we fully achieve the third approach with a Si semiconductor-electrocatalyst system exhibiting strong electronic coupling by leveraging the well-studied cobaloxime HER catalyst and chemisorbing it onto Si NCs via an ethylenepyridine molecular tether. We characterize the hybrid system using steady-state photoluminescence (PL), UV–vis absorption and transient absorption (TA) spectroscopies, CV, spectroelectrochemistry (SEC), electron paramagnetic resonance (EPR) spectroscopy, and DFT calculations. These results reveal that a new electronic structure arises from hybridization between high-energy Si NC band states and symmetry-matched cobaloxime molecular orbitals. We anticipate this work will provide a paradigm shift in the community’s ability to heterogenize molecular catalysts to surfaces and control the energetics of PEC solar fuel assemblies and related systems.

## Results and Discussion

### Cobaloxime-Si NC Hybrid Synthesis

Cobaloxime-Si NC hybrid systems are synthesized by leveraging cobaloxime’s affinity for binding an axial pyridyl (py) ligand.^33–37^ The first hybrid system is based on the two-step method we previously developed for tethering a Re-tricarbonyl aldehyde-appended bipyridine ligand to a Si NC surface.^19^ As shown in Figure 1a, hydrogen-terminated Si NCs formed from plasma-enhanced chemical vapor deposition (PECVD) from silane gas are undersaturated with dodecyl ligands via a radical reaction in neat 1-dodecene to provide Si–C_12_ NCs. Subsequent reaction with 4-formylpyridine (fpy) provides pyridyl-functionalized NCs where the pyridyl group is appended to the Si NC surface via a methylene ether linkage, Si-OCH_2_–py, hereafter referred to as Si-fpy based on the starting 4-formylpyridine for simplicity (Figure 1c). The separation of C_12_- and fpy-functionalization steps prevents surface saturation with fpy groups, resulting in poor colloidal stability, which would otherwise occur due to the 2 orders of magnitude faster kinetics of silicon-based radical attack on formyl arenes versus primary alkenes.^38,39^

**Figure 1:**
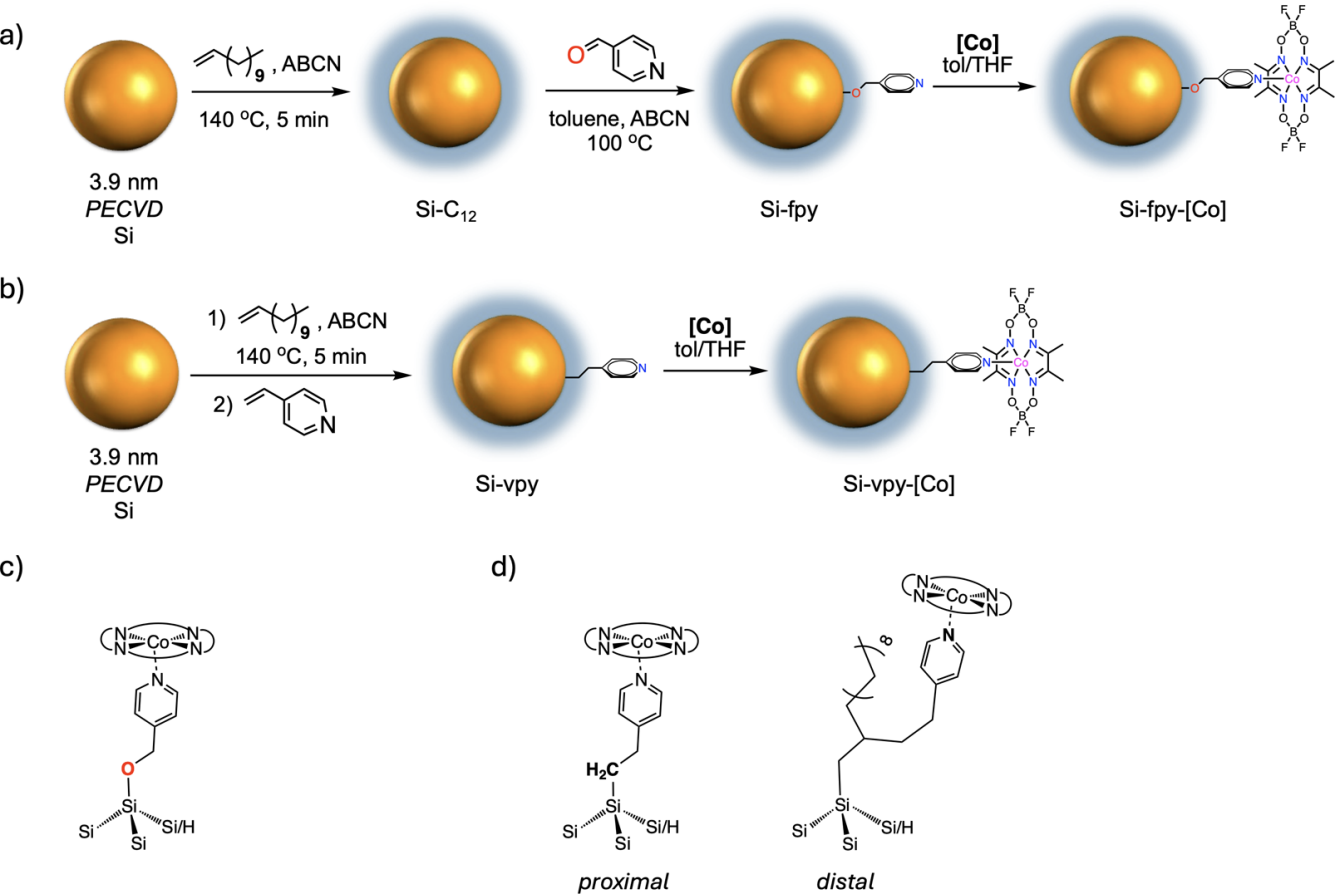
(a) Stepwise synthesis of Si-fpy from 4-formylpyridine and its reaction with [Co]. (b) One-pot synthesis of Si-vpy from 4-vinylpyridine and its reaction with [Co]. Binding orientations of [Co] in (c) Si-fpy-[Co] and (d) Si-vpy-[Co] in its proximal and distal configurations.

The second approach, displayed in Figure 1b, involves a one-pot functionalization of Si NCs with both 1-dodecene and 4-vinylpyridine (vpy) via radical-initiated hydrosilylation to generate Si NCs where the pyridyl group is appended to the Si NC surface via an ethylene bridge, Si-CH_2_CH_2_–py, hereafter simply referred to as Si-vpy (Figure 1d). Successful incorporation of pyridyl groups in both Si-fpy and Si-vpy is evident from diffuse reflectance infrared Fourier transform spectroscopy (DRIFTS) of their thin films, which both display aromatic C=C/C=N vibrational modes at ca. 1600 and 1550 cm^–1^ (Figure S1). Comparing the DRIFTS data of the two samples suggests that Si-vpy from the 1-pot approach contains a slightly higher pyridyl density on the surface compared with Si-fpy from the 2-step method (Figure S1c), which we explain by two reasons. First, Si–C_12_ NCs from the two-step method have fewer reactive surface hydrides following undersaturation with the initial dodecyl ligand shell, leading to a slightly lower fpy coverage compared to vpy. Second, the 1-pot approach affords the possibility of introducing additional pyridyl groups at the β-position of the dodecyl chain (Figure 1d), resulting from the radical chain reactions as laid out in the original mechanistic study by our group.^40^ We term this second orientation “distal” to distinguish from the one directly bound to the Si surface that we term “proximal” taking inspiration from the enzyme literature.

After pyridyl incorporation, both Si-fpy and Si-vpy exhibit improved dispersibility in polar solvents, such as tetrahydrofuran (THF). As such, subsequent studies are performed in a mixture of solvents, where both the cobaloxime catalyst and pyridine-functionalized Si NCs are soluble. Initial attempts in binding the classical cobaloxime catalyst featuring glyoxime ligands were unsuccessful, which we ascribe to the bridging protons in the ligand backbone. Synthesizing the difluoroboryl-dimethylglyoxime catalyst Co­(dmgBF_2_)_2_(L)_2_ (L = solvent molecule),^41,42^ hereafter referred to simply as [Co], and adding this complex to pyridyl-functionalized Si NC colloids in THF/toluene (tol) solvent mixtures results in spontaneous chemisorption via open axial sites on the [Co] complex. We also find changes to some of the properties within the Si-vpy-[Co] hybrid system with gentle heating to 50 °C over 12 h. We posit that the initial [Co] binding with Si-vpy results in a portion of [Co] attachment via distal pyridyl groups that positions some [Co] units within spatial proximity for energy transfer but does not result in strong electronic coupling that occurs only through direct tethering to the Si surface via proximal pyridyl groups, as will be discussed in detail below.

### Steady-State Photoluminescence (PL) and UV–vis Absorption Spectroscopies

The spontaneous reaction between pyridyl-functionalized Si NCs Si-fpy and Si-vpy with the [Co] complex is easily monitored by changes in their emission and absorption spectra. Prior to adding [Co], Si–C_12_, Si-fpy, and Si-vpy all display intense band edge emission centered around 825 nm and weak surface oxide state-related defect emission at ∼475 nm,^43^ consistent with our prior reports of 3.9 nm diameter Si NCs fully saturated with long-chain alkyl and alkoxyl ligands.^40,44^ As [Co] is added, decreases in PL peak intensities are observed (Figure 2a–c), suggesting Si NC emission quenching by interaction with [Co]. Plots of the relative PL peak intensities *I*/*I*_0_ (%) versus [Co] equivalents show that a slight PL intensity decrease to ca. 85% at up to 30 equiv [Co] is found for Si–C_12_ (Figure S2a), which we attribute to energy transfer within an associative complex Si–C_12_ + [Co] similar to what was found for Re­(bpy)­(CO)_3_Br.^20^ More significant drops are observed for the pyridyl-containing Si NCs for which the PL peak energies of Si-fpy and Si-vpy decrease to their minimum *I*/*I*_0_ ratios of 35% at ∼15 equiv and 12% at ∼20 equiv of [Co], respectively (Figure S2b-c). Higher [Co] concentrations and mild heating (50 °C, 12 h) had no effect on the PL intensity, suggesting that these [Co] equivalencies represent the saturation values for surface adsorption. The slightly higher [Co] equiv needed for Si-vpy to reach its maximum quenching compared to Si-fpy is consistent with our observation from DRIFTS that the one-pot synthesis results in a slightly higher pyridyl coverage than the 2-step method.

**Figure 2:**
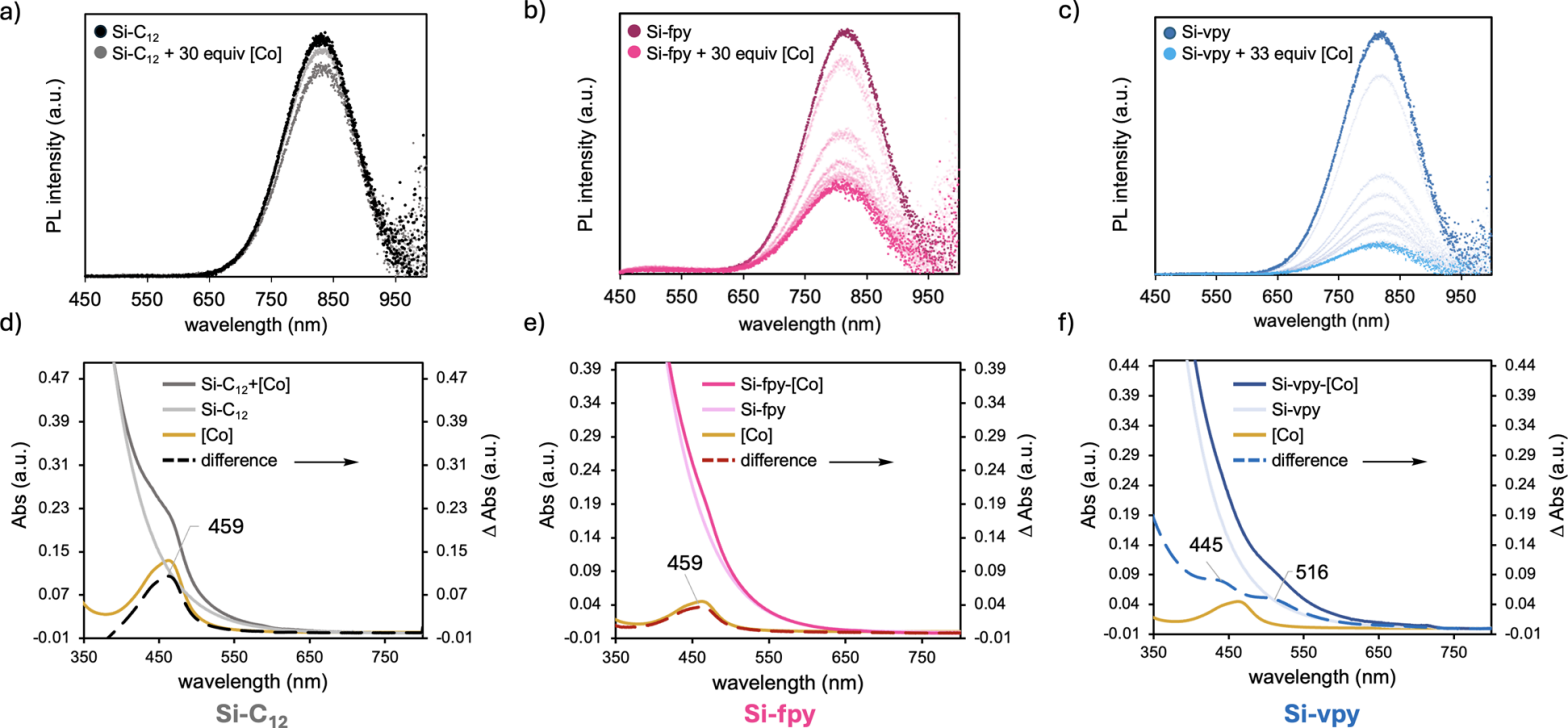
(a–c) Steady-state PL spectra for Si–C_12_, Si-fpy, and Si-vpy at different equivalents of [Co] excited at 405 nm. (d–f) Absorption spectra (left axes) with difference spectra (ΔAbs) plotted as dashed lines (right axes) conducted with [Co] near the saturation limits. All spectra are acquired in 3:1 THF/tol (v/v) solvent mixtures.

We examine the quenching behavior in more detail by plotting *I*_0_/*I* vs [Co] equiv (Figure S2d–f) from which it is found that the quenching profiles of both Si-fpy and Si-vpy mixed with [Co] deviate significantly from the Stern–Volmer linear relationship for collisional (dynamic) quenching.^45,46^ Instead, the PL quenching observed here is consistent with static quenching within an association complex in which the quencher molecules are located inside an interacting sphere surrounding the emitter, where they would have little or no quenching outside this interacting sphere.^47–50^ Further evidence of such an interaction quenching model is found in the *I*_0_/*I* vs [Co] equiv plot for Si–C_12_ + [Co] (Figure S2d–f), where minimal change to the PL intensity is observed with increasing [Co] equiv, owing to the weak interactions expected between the Si–C_12_ surface and [Co]. From these PL data, we conclude that [Co] is chemisorbed to surface-bound pyridyl groups—which we term Si-fpy-[Co] and Si-vpy-[Co] to distinguish from the physical mixture Si–C_12_ + [Co]—that introduces nonradiative pathways for Si NC excited-state relaxation.

Next, we find stark differences in the absorbance spectra of these samples that provide the first evidence of hybridized states in Si-vpy-[Co]. Figure 2d–f displays the absorbance spectra of Si–C_12_, Si-fpy, and Si-vpy before and after adding 15 and 20 equiv of [Co], respectively, near the saturation limit to avoid any free [Co] that would complicate the absorbance experiments. Since Si NC absorption dominates the spectra, we plot the difference spectra (dashed lines) on the secondary axis to highlight the features from [Co]. Both the Si–C_12_ + [Co] and Si-fpy-[Co] difference spectra (Figure 2d–e) closely match the [Co] absorption (gold line), in which the only feature present is the [Co] main absorption at 459 nm. This finding indicates that the spectra are superpositions of the [Co] absorption onto the Si NC exponential absorptions, suggesting that there is no change in the Si or [Co] electronic structures in these two cases. In contrast, the difference spectrum of Si-vpy-[Co] (Figure 2f) reveals two new absorption features at 445 and 516 nm that we assign to new electronic transitions within Si-vpy-[Co]. The similar intensities and positions of these new peaks compared to the free [Co] absorption at 459 nm suggest that these are [Co]-based transitions that are perturbed by interactions with Si. A second possibility giving rise to these new absorption features is a change to the local environment of [Co] from pyridyl coordination that encompasses [Co] within the hydrophobic ligand matrix. We find this possibility unlikely since a single new absorption feature resulting from a hypsochromic (blue) shift should be observed upon [Co] moving from the THF/tol solvent environment to a hydrophobic one within the ligand matrix, whereas two new features are found. A third possible reason for the new absorption features is [Co] aggregation on the Si NC surface, which also is unlikely based on the subsaturated [Co] concentration relative to pyridyl binding groups that minimizes the potential for aggregation.

These absorbance data combined with the higher PL quenching efficiency cf. Si-fpy-[Co] indicates strong electronic communication between Si and [Co] within the Si-vpy-[Co] hybrid system. Meanwhile, Si-fpy-[Co] seems to be an uncoupled system in which [Co] is chemically bound to the Si NC but participates in energy transfer only due to its spatial proximity inside the interacting sphere. These data suggest that the ether-O atom in the methylene ether linkage in Si-fpy-[Co] does not enable strong electronic coupling relative to the methylene group in the ethylene linkage in Si-vpy-[Co]. This result also is surprising given prior reports showing that hybridized states between Si NCs and conjugated organics require an unsaturated vinyl linker Si—CH=CH—R (R = conjugated organic),^51–53^ not a saturated ethylene bridge as found here, and is rationalized from DFT calculations, vide infra.

### Transient Absorption Spectroscopy (TAS) and Spectroelectrochemistry (SEC)

We next probed the photodynamics within these Si NC-electrocatalyst systems using TAS to better understand the possible PL quenching mechanisms and the nature of the strong electronic coupling within Si-vpy-[Co]. In Figure 3a–c, we compare the TAS data of the physical mixture of Si–C_12_ + [Co], Si-fpy-[Co], and Si-vpy-[Co] at the [Co] saturation limits. All samples exhibit a broad photoinduced absorption (PIA) upon photoexcitation at 400 nm that spans the visible probe window assigned to the Si NC along with a sharp decrease in amplitude at 450 nm at early pump–probe delay times (250 fs) assigned to the [Co] ground state bleach (GSB) superimposed on top of the broad photoinduced absorption from the Si NCs (assignments from TAS spectra of Si–C_12_ and [Co] alone, Figure S3). The predominant difference in these spectra is the peak centered at 640 nm present in the Si-vpy-[Co] spectrum (Figure 3c) that is absent in the Si–C_12_ + [Co] and Si-fpy-[Co] spectra (Figure 3a-b). This feature is also absent in the TA spectrum of the free [Co] complex excited at the same wavelength (Figure S3b), indicating that this new feature comes from a strong interaction within Si-vpy-[Co]. We note that the 640 nm feature at 250 fs is also observed at a similar amplitude in a freshly mixed Si-vpy-[Co] sample (Figure S4) relative to the sample undergoing gentle heating shown in Figure 3c, consistent with PL data that also does not change with heating at the [Co] saturation limit.

**Figure 3:**
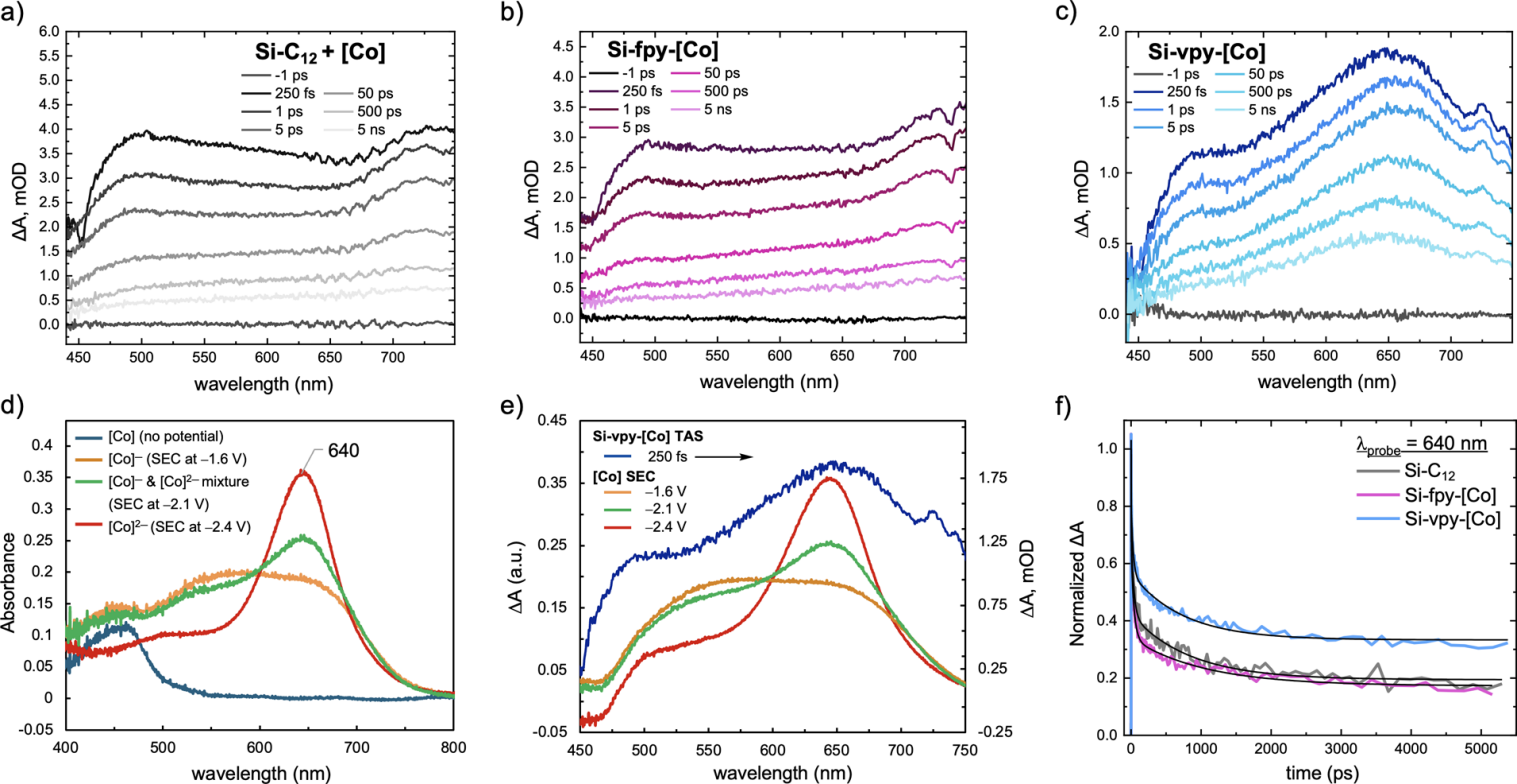
(a–c) TAS data of Si–C_12_ + [Co], Si-fpy-[Co], and Si-vpy-[Co] in 3:1 THF/toluene solvent mixture, photoexcited at 400 nm at the [Co] saturation limits derived from PL data and heated to 50 °C for 12 h to ensure saturation. (d) Steady-state absorbance spectra of [Co] at different redox states from SEC measurements in 3:1 THF/tol (v/v) solvent mixture. (e) Difference spectra of [Co] SEC data (obtained by subtracting out the [Co] absorption at no potential applied) overlaid with Si-vpy-[Co] TAS data at 250 fs. (f) Normalized kinetic decay of TAS at λ = 640 nm.

We next perform spectroelectrochemical (SEC) measurements of the free [Co] complex to identify the new 640 nm feature in TAS. In Figure 3d, we plot SEC data as absorbance spectra acquired at no applied potential, −1.6 V, and −2.4 V vs Fc^+/0^ that are assigned to the [Co^II^]^0^, [Co^I^]^−^, and [Co^0^]^2–^ complexes, respectively (see the electrochemical section). Additionally, we also plot the absorbance spectrum acquired at −2.1 V versus Fc^+/0^ that effectively generates an equimolar mixture of [Co^I^]^−^ and [Co^0^]^2–^ (Figure 3d, green trace). In Figure 3e, these [Co] SEC data are plotted as difference spectra and overlaid with the earliest time TA spectrum for Si-vpy-[Co]. Astoundingly, the feature observed at 640 nm in TAS appears only at strongly cathodic electrochemical potentials, −2.1 V vs Fc^+/0^ and above, where the [Co] complex is reduced from [Co^I^]^−^ to [Co^0^]^2–^. The broadened 640 nm feature in TAS mimics the line shape of the SEC data at −2.1 V, suggesting that it stems from a mixture of singly and doubly reduced [Co]-centered species upon photoexcitation of Si-vpy-[Co]. Given the Si conduction band edge energy is far too low to induce CT into the high-lying unoccupied molecular orbitals of the transition metal catalyst,^20^ it is likely that the doubly reduced [Co^0^]^2–^ is generated from multiple hot electron transfer events from Si-based states into states related to [Co]. These PIA events are not observed in Si-fpy-[Co] and presumably are enabled by strong electronic coupling within Si-vpy-[Co]. Lastly, another exciting observation is found in the kinetic decay data at λ_probe_ = 640 nm that shows the Si-vpy-[Co] spectrum has ∼20% more amplitude at 5 ns cf. that of Si–C_12_ or Si-fpy-[Co], which suggests that both the singly [Co^I^]^−^ and doubly reduced [Co^0^]^2–^ related states are long-lived, as would be necessary in any practical PEC or photocatalytic system. The surprisingly long lifetime of photogenerated charges observed here can be rationalized from the DFT calculations vide infra.

### Electrochemistry, EPR, and SEC of Colloidal Si-vpy-[Co]

We further characterized the unique Si-vpy-[Co] system using electrochemistry, EPR, and SEC analysis. All experiments were conducted as colloids. First, the electrochemical response of free [Co] features a broad first redox wave and a more defined second wave with a classical diffusion-limited shape (Figure 4a, top panel). We posit that its broadened first redox wave in a THF/toluene mixture is due to the presence of multiple [Co] species weakly coordinated by different numbers of THF molecules, which can be 0, 1, or 2. Next, we find that the CV of [Co] changes dramatically immediately after mixing with Si-vpy (for the CV of Si-vpy alone, see Figure S5). The first wave ascribed to [Co]^0/–^ in the Si-vpy-[Co] CV is sharp, and the second wave disappears entirely (Figure 4a, middle panel) compared with the CV of free [Co]. We attribute the narrowing of the first wave to binding to surface-bound pyridyl groups and the suppression of the second wave to multiple pyridyl group binding events to open axial sites on [Co]. A control experiment corroborates this latter hypothesis in which adding 3 equiv of vpy to free [Co] results in near complete suppression of the [Co]^−/2–^ reduction wave (Figure S6a,b). This is possible in Si-vpy-[Co] due to Si-vpy from the 1-step method containing distal pyridyl binding sites that can participate in bis-coordination to both axial faces of [Co] (Figure 4b, top structure).

**Figure 4:**
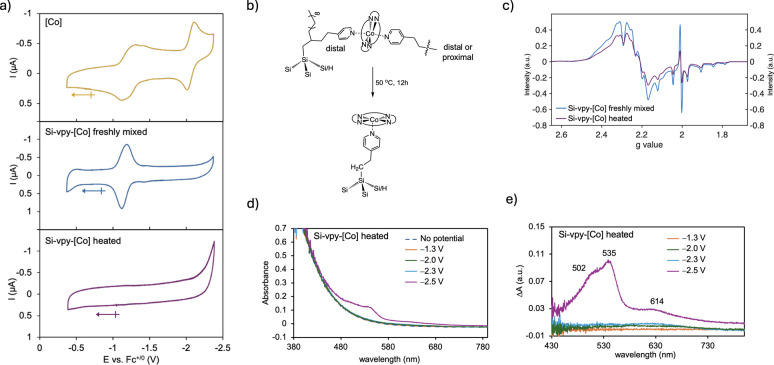
(a) CVs of 25 μM [Co] in THF/tol 3:1 (top, gold), 25 μM Si-vpy with 25 μM [Co] freshly mixed (middle, blue), and after 12 h heating at 50 °C (bottom, purple) (conditions: GCE electrodes, 50 mM TBAPF_6_, and 50 mV/s scan rate). (b) Hypothesized reorganization of the binding motif in Si-vpy-[Co] at ∼1 equiv [Co] upon heating. (c) Overlaid EPR spectra of Si-vpy-[Co] at its saturation limit freshly mixed and heated. (d) SEC spectra of Si-vpy-[Co] with 1 equiv [Co] after heating to 50 °C for 12 h. (e) Difference SEC spectra from (d) by subtracting absorption from Si-vpy-[Co] at no applied potential.

The CV changes again following heating Si-vpy-[Co] into a featureless CV where both the first and second redox waves are not observed, and a sharp increase in current begins at −2.3 V (Figure 4a, bottom panel). We attribute the disappearance of all redox features to the reorganization of the [Co] binding motif, where a transition to a proximal-distal configuration occurs upon mild heating of Si-vpy-[Co] at this low [Co] concentration (Figure 4b). The absence of redox features is reminiscent of the electronic coupling in graphite-conjugated catalysts pioneered by Surendranath and coworkers described previously^21–24^ and, to our knowledge, is the first observation of this behavior in a semiconductor-molecular catalyst system. EPR spectroscopy experiments confirm changes to the [Co] electronic structure via interaction with Si before and after heating Si-vpy-[Co], consistent with these CV results. As shown in Figure 4c, heating freshly mixed Si-vpy-[Co] (20 equiv [Co]) both decreases the intensity of the Co^II^ lines and broadens the [Co] hyperfine structure, indicating strong hyperfine interaction with surface ^29^Si nuclei. Full discussion of the EPR data is included in the SI (Figure S7 and associated text).

In contrast to the Si-vpy-[Co] CV data with 1 equiv [Co], we do not find suppression of the [Co] redox features in Si-fpy-[Co] CVs at 1 equiv [Co] either before or after heating (Figure S8a,b). This result is consistent with optical spectroscopy measurements, where Si-fpy-[Co] behaves as a physical mixture of electronically uncoupled species. Full electrochemical analysis and insight into the binding motifs at both low and high [Co] concentrations as well as further support for hybridized states in Si-vpy-[Co] from CV analysis at strongly cathodic potentials are provided in the SI (Figure S8–S11 and associated text).

We rationalize these observations by considering the fundamentals of electrochemical charge transfer. In brief, suppression of a redox wave generally stems from burying the electrochemically transferred charge within the electrochemical double layer that screens charge-balancing counterions from accommodating the charge transfer event into a localized state.^21–24^ Since redox wave suppression does not occur in the Si-fpy-[Co] system that exclusively features proximal fpy binding, this implies that [Co] binding at proximal fpy sites features localized redox states that interact strongly with charge-balancing counterions. Thus, it would be reasonable to assume that [Co] also is ineffectively screened and should exhibit redox features upon binding at similar proximal vpy sites in Si-vpy-[Co]. Yet, clear evidence of charge screening is found in the Si-vpy-[Co] CV data following heating (Figure 4a, bottom panel).

We hypothesize that electrochemically transferred charge to Si-vpy-[Co] does not reside in local states within [Co]—where it would be accessible to charge-balancing counterions in the electrolyte—but instead is delocalized throughout the entire Si-vpy-[Co] system. Indeed, DFT calculations find several high-energy electronic states with electron density probabilities exhibiting charge delocalization in Si-vpy-[Co], vide infra. Lastly, spectroelectrochemical (SEC) analysis provides evidence of significant charge delocalization in Si-vpy-[Co]. As shown in Figure 4d,e, discrete [Co]-centered optical transitions are only found following chronoamperometry at −2.5 V, well above the [Co] first and second reduction potentials. The fact that the charge passed from chronoamperometry below this extreme cathodic potential does not result in any discrete molecular [Co] optical transitions is strong evidence that electrochemically transferred charge is delocalized over the entire Si-vpy-[Co] system. See SI, Figure S12, and associated text for a full discussion of SEC data.

### Density Functional Theory (DFT) Calculations

To better understand the origins of the optical and electrochemical behavior observed in Si-vpy-[Co], we performed DFT calculations to compare the Si-vpy-[Co] and Si-fpy-[Co] systems. Calculations were carried out using ORCA (version 5.0.3) with the M06-L meta-GGA functional and triple-ζ def2-TZVP basis set for all atoms, and visualizations were created using VESTA.^54–57^ The simulated Si NC size was limited to 2.0 nm, as larger sizes (2.5 and 3.0 nm) did not converge. Additionally, hydride surface termination was employed instead of a hydrocarbon to reduce computational cost. The energetic differences between hydride and hydrocarbon termination were detailed in our previous publication,^20^ and these are not expected to impact the results here, focusing on the energetics resulting from fpy versus vpy surface linkers. Finally, calculations were conducted with one methylene ether pyridine (fpy) or one ethylene pyridine (vpy) linkage attached to 1 equiv [Co], mimicking the experimental conditions used for CV data in Figure 4a. Additional computational details, including structure generation, calculation parameters, and population analysis procedures (discussed below), are provided in the SI.

Mulliken population analysis was used to identify hybridized molecular orbitals (MOs) having significant contributions from both Si and [Co]. Given the much larger number of atoms in Si NCs compared to that in [Co], we set the minimum threshold for hybridization at 3% [Co] contribution. Surprisingly, we find no evidence of electronic hybridization in either Si-fpy-[Co] or Si-vpy-[Co] in the orbitals near the frontier MO region, ranging from HOMO-10 to LUMO+10. All states in this region involve either Si band edge states or [Co] d-orbitals. Hybridization is observed only when surveying a wider range of states (HOMO-100 to LUMO+100) corresponding to new hybridized orbitals derived from deep Si band states and high-energy [Co] orbitals, as shown in the energy diagrams in Figures S13 and S14. Though we find some differences between Si-vpy-[Co] and Si-fpy-[Co] in both energy levels and orbital ordering, hybridized states are found for both Si-vpy-[Co] and Si-fpy-[Co]. What, then, is the origin of the starkly different behavior in these systems?

The key mechanistic insight explaining the optical and electrochemical behavior observed in Si-vpy-[Co] is revealed through close inspection of the bonding character between the Si NC and the linker attachment chemistry in many hybridized states within Si-vpy-[Co] and Si-fpy-[Co]. Figure 5 displays MO visualizations for one such state, illustrating the spatial distribution and phase of the wave function for the identical occupied MO 1474α (HOMO-61α) in the Si-vpy-[Co] and Si-fpy-[Co] systems, derived from the same Si NC and [Co] MOs that facilitates a direct comparison. Focusing on the atoms attached to the Si NC surface, we find that Si-vpy-[Co] features σ-bonding character at the *Si–C bond (where *Si denotes a surface Si atom) as well as at the sp^3^-sp^3^ C–C bond across the *Si–CH_2_–CH_2_– ethylene bridge (Figure 5a). In stark contrast, π-antibonding character dominates the *Si–O and O–C bonds across the methylene ether bridge *Si–O–CH_2_– in Si-fpy-[Co]. These observations suggest that strong electronic coupling is allowed in Si-vpy-[Co], whereas for Si-fpy-[Co], it is disrupted because of antibonding character at the surface chemistry linkage derived from the O atom in the methylene ether tether, even in the bonding (occupied) MO 1474α (HOMO-61α). Figures S15 and S16 present other examples of this contrasting σ-bonding and π-antibonding character in Si-vpy-[Co] and Si-fpy-[Co], respectively, for several other hybridized bonding orbitals (occupied states) and in one high-energy unoccupied state. We hypothesize that this difference originates from the highly electronegative O atom, which likely exhibits σ-bonding character only with much deeper Si valence band states that are outside the optical and electrochemical energy regions explored here (i.e., below HOMO-100).

**Figure 5:**
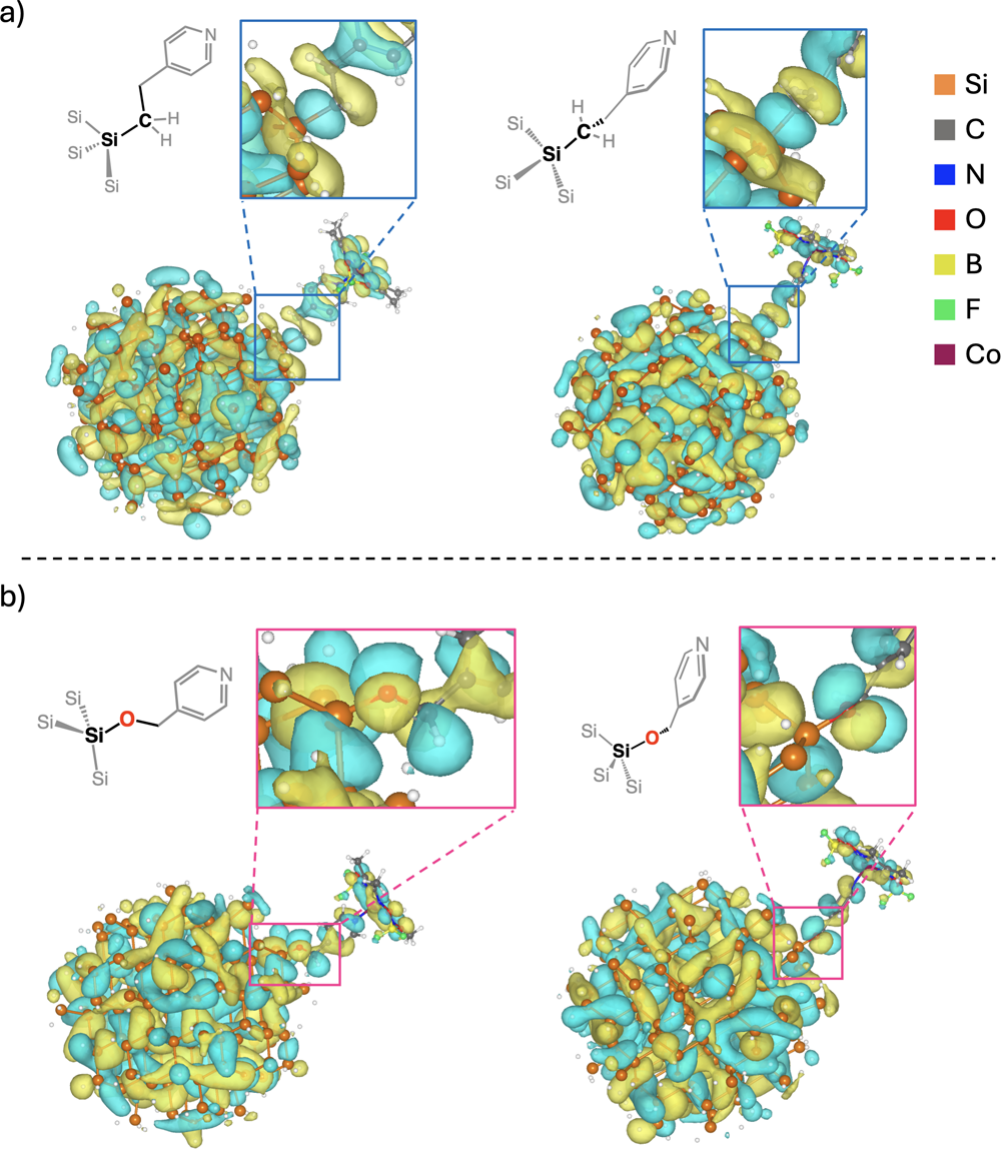
(a) Visualization of MO 1474α (HOMO-61α) in Si-vpy-[Co] in two different perspectives with the zoomed-in view focusing on the electron probability density on the *Si–CH_2_–CH_2_– linker. (b) Visualization of MO 1474α (HOMO-61α) in Si-fpy-[Co] in two different perspectives with the zoomed-in view focusing on the electron probability density on the *Si–O–CH_2_– linker. Molecular structures are shown in the same orientation as the zoomed-in insets to aid visualization.

Lastly, we consider the differences between these DFT calculations and the systems used for optical, electrochemical, and EPR experiments. Certainly, we expect discrepancies in the absolute values of the hybridized state energies found in the computational model (2.0 nm diameter H-terminated Si NC) and those in the actual system (3.9 nm with C_12_ termination). We posit that the calculations are true to the less quantum-confined system in that hybridized states likely do not exist at the HOMO/LUMO region but instead are derived from deeper energetic states. We first point back to the two new high-energy transitions observed in the absorbance spectra in Figure 2f that indicate hybridized states at 2.4 and 2.8 eV (516 and 445 nm) in the 3.9 nm Si-vpy-[Co] system. Second, the TAS data in Figure 3f show an exceptionally long lifetime (>5 ns) for high-energy photogenerated charge carriers within Si-vpy-[Co]. We would anticipate that 5 ns should be sufficient time for thermalization from high energy into low-energy states, where rapid recombination should result. Yet, this is not what is found from the TAS data, and instead, a mixture of high-energy species with character from singly reduced [Co^I^]^−^ and doubly reduced [Co^0^]^2–^ persists >5 ns. This seems to imply that photoexcitation occurs from Si valence band states into high-energy Si-vpy-[Co] hybridized states where hot electrons reside within hybridized excited state MOs and/or thermally relax into [Co]-centered MOs. In either case, recombination is mitigated because holes remain localized in Si valence band states. Lastly, our extensive CV and SEC analyses support the existence of high-energy hybridized states where charge passed into Si-vpy-[Co] up to −2.3 V vs Fc^+/0^ does not reside on localized [Co] states but instead appears to be delocalized over the entire Si-vpy-[Co] system. Thus, we conclude that the systems investigated with optical and electrochemical experiments are consistent with hybridized states derived from deep Si band states and high-energy [Co] MOs beyond the frontier HOMO-10 to LUMO+10 region.

## Conclusion

The discovery of hybridization between deep semiconductor band states and high-lying molecular orbitals in surface-tethered catalysts has broad implications for the design of solar fuel systems based on direct PEC conversion of light energy into chemical energy. The attachment chemistry appears to be the critical factor necessary to achieve hybridization, where we showed that the molecular group (ethylene versus methylene ether) linking Si and the pyridine tether dictates whether the pyridyl-appended molecular catalyst hybridizes with the Si or not. This extreme sensitivity to the linking group chemistry teaches us that it is insufficient to simply provide a spatial proximity between a semiconductor and a surface-bound catalyst to achieve efficient photoinduced processes. In addition to the atomic chemistry at the interface, a convolution of complex parameters such as how the atomic matrix environment surrounding the semiconductor manipulates its energetics as we and others have discussed previously^44,58–60^ as well as how the molecular environment enables the tethering group and catalyst to achieve the right symmetry^61^ also appear to play key roles in enabling the creation of hybridized states with strong electronic coupling. Finally, the observation of long-lived hot electrons in Si-vpy-[Co] opens the door to the investigation of hybrid semiconductor PEC systems with high redox potential molecular catalysts such as those being used to generate chemical fuels and other energy-rich chemicals. Rational synthesis of such hybrid schemes must consider the multitude of factors that determine whether the system will achieve strong electronic coupling, especially the surface attachment chemistry employed to heterogenize the molecular catalyst.

## Supplementary Material





## Notes

Mulliken population analysis tables for HOMO-100 to LUMO+100 for Si-fpy-[Co] and Si-vpy-[Co] (XLSX)
Experimental details; DRIFT spectra; PL *I*_0_/*I* vs [Co] equiv plots; TAS spectra of Si–C_12_ and [Co] only and Si-vpy-[Co] freshly mixed and heated; CVs of Si–C_12_, Si-vpy, and [Co] with different vpy equiv; EPR analysis and spectra of Si-vpy, free [Co], and Si-vpy-[Co] at different [Co] equiv; CV analyses for [Co] binding environment in Si-vpy-[Co] and Si-fpy-[Co], Si-fpy-[Co] freshly mixed and heated, and Si-vpy-[Co] at 20 equiv [Co] freshly mixed and heated; different binding motifs at initial binding and following heating; CVs of Si-vpy-[Co] in different solvent ratios; analysis of CVs in the high potential region; CVs of Si-vpy and Si-vpy-[Co] freshly mixed and heated at high cathodic potential; SEC analysis; SEC data of [Co] and Si-vpy-[Co]; computational details; energy diagrams of Si-vpy-[Co] and Si-fpy-[Co] from HOMO-100 to LUMO+100; and visualizations of selected hybridized MOs in Si-vpy-[Co] and Si-fpy-[Co] (PDF)
