# Supplementary material for: High-Energy Hybridized States Enable Long-Lived Hot Electrons in Cobaloxime-Silicon Nanocrystal System
Source: J Am Chem Soc. 2026 Feb 9;148(6):6412–21. doi: 10.1021/jacs.5c19326 (PMC12921872; doi:10.1021/jacs.5c19326)
Supplement: Supplementary file 2 [file ja5c19326_si_002.pdf]

Supporting Information for:

High-Energy Hybridized States Enable Long-Lived Hot Electrons in Cobaloxime-Silicon Nanocrystal System

Trung H. Le,[a] Melissa K. Gish,[a] Simran S. Saund,[a] Taylor Aubry,[a] and Nathan R. Neale*[a,b]

[a]Materials, Chemical, and Computational Science Directorate, National Laboratory of the Rockies, Golden, Colorado 80401, United States

[b]Renewable and Sustainable Energy Institute, University of Colorado Boulder, Boulder, Colorado 80309, United States

Table of contents

I.	Experimental Details	S-2
II.	Supporting data	S-6
a.	Figure S1. DRIFTs spectra	S-6
b.	Figure S2. PL intensity vs. [Co] equiv plots	S-7
c.	Figure S3. TAS of Si-C_{12} and [Co] only	S-7
d.	Figure S4. TAS of Si-vpy-[Co] freshly mixed and heated	S-8
e.	Figure S5. CVs of Si-C_{12} and Si-vpy	S-8
f.	Figure S6. CVs of [Co] with different vpy equiv	S-9
g.	EPR analysis	S-9
h.	Figure S7. EPR spectra of Si-vpy, free [Co] and Si-vpy-[Co] at different [Co] equiv	S-10
i.	CV analyses for [Co] binding environment in Si-vpy-[Co] and Si-fpy-[Co]	S-10
j.	Figure S8. CVs Si-fpy-[Co] freshly mixed and heated and Si-vpy-[Co] at 20 equiv [Co] freshly mixed and heated	S-11
k.	Figure S9. Different binding motifs at initial binding and following heating.	S-12
l.	Figure S10. CVs of Si-vpy-[Co] in different solvent ratios	S-13

m. Analysis of CVs in the high potential region S-14

n. Figure S11. CVs of Si-vpy and Si-vpy-[Co] freshly mixed and heated at high cathodic potential S-14

o. SEC analysis S-15

p. Figure S12. SEC data of [Co] and Si-vpy-[Co] S-16

III. Computational Details S-17

q. Figure S13. Energy diagram of Si-vpy-[Co] MOs from HOMO-100 to LUMO+100 S-18

r. Figure S14. Energy diagram of Si-fpy-[Co] MOs from HOMO-100 to LUMO+100 S-19

s. Figure S15. Visualization of selected hybridized MOs in Si-vpy-[Co] S-20

t. Figure S16. Visualization of selected hybridized MOs in Si-fpy-[Co] S-20

IV. References S-21

I. **Experimental** Details.

General.

All air-free reactions and manipulations were performed using standard Schlenk-line and syringe/rubber septa techniques under N_2 or in Ar atmosphere gloveboxes. Dry solvents were distilled over Na/benzophenone (THF and toluene) or CaH_2 (MeCN, and 1-dodecene). Boron trifluoride diethyl etherate $BF_3{\cdot}Et_2O$ was freshly distilled before use to make the $Co(dmgBF_2)_2$ compound synthesized based on reported procedure with a slight modification.[1,2] Other reagents were purchased from commercial sources and dried over P_2O_5 before use. 3.9 nm intrinsic Si NCs were grown by PECVD as reported previously.[3,4] Si-C_{12} was synthesized according to our previously reported procedures.[3–5]

Infrared spectroscopy.

Infrared spectra were collected on Bruker Alpha II FTIR spectrometer housed in an argon-filled glovebox and fitted with either a diffuse reflectance Fourier transform spectroscopy (DRIFTS) or a diamond attenuated total reflectance (ATR) module. DRIFTS samples were acquired by either drop-casting an analyte onto an Au-coated reflective silicon wafer

substrate or directly depositing the powder analyte onto the same substrate. ATR measurements were acquired by directly clamping the powder sample onto the ATR prism.

Photoluminescence (PL) spectroscopy.

Emission spectra were acquired on solution phase samples excited by a 1.4 A ThorLabs LED. The excitation source was filtered through a 450 nm shortpass filter and 410 nm bandpass filter and delivered to the sample by fiberoptic. The emitted light was collected at 0° from the excitation source and filtered through a 450 nm longpass filter before being detected an OceanFX UV-vis detector and NIRQuest near IR detector.

UV-Vis spectroscopy.

Steady state UV-vis spectra were collected on samples in a 2 mm path length quartz cuvette with either a CARY-5000 or CARY-7000 spectrophotometer.

Transient absorption spectroscopy (TAS).

Transient absorption data were collected using a Ti:Sapphire regenerative amplifier with an 800 nm fundamental (1 kHz, 90 fs pulse width) (Coherent Astrella). Pump and probe beam paths were created by splitting the beam at the outset. The 400 nm pump (500 uW) was generated in an optical parametric amplifier (TOPAS, Light Conversion) and chopped at 500 Hz to modulate the pump on and off. The probe pulse traveled through a multi-pass mechanical delay stage and focused through a thin sapphire crystal to generate a white light supercontinuum (λ_{probe} = 440-850 nm). The pump and probe were focused and spatially overlapped at the sample. Signal-to-noise ratio was maximized by picking off a small portion of the probe before the sample to act as a reference. Changes in the probe spectrum were monitored by a fiber-coupled multichannel spectrometer with a CMOS sensor. Data were collected and chirp corrected using Helios and Surface Xplorer programs (Ultrafast Systems), respectively. Data were analyzed and plotted in Origin (OriginLabs).

Electrochemistry.

All electrochemical measurements were taken in an argon-filled glovebox using a Biologic SP- 300 potentiostat. Colloidal CVs were taken in 3:1 THF/toluene solvent mixture with 50 mM $TBAPF_6$ supporting electrolyte and at 50 mV/s scan rate. Glassy carbon electrodes (GCE, 0.3 cm diameter) were polished on a polishing pad coated wet with 0.05 μm alumina slurry followed by rinsing with water and methanol. Sonication was avoided to protect the glassy carbon – metal pin connection. Thin-films were prepared by drop-casting the NC colloids onto the inverted electrode and letting them dry in the glovebox at ambient temperature. All CVs were taken in a three-electrode setup with a platinum wire counter electrode and a silver wire pseudo reference electrode chambered with the same $TBAPF_6$

electrolyte solution as the bulk electrolyte and separated from the bulk by a CoralPor® frit. Solution resistance was corrected for *in situ*. The potentials are reported versus $Fc^{+/0}$ by adding ferrocence at the end of each experiment batch to calibrate the pseudo reference electrode.

Spectroelectrochemistry (SEC).

SEC was performed with colloidal Si NCs samples in a 1.7 mm pathlength quartz cuvette with a 1 cm x 1 cm upper well using an Au-coated ceramic honeycomb electrode card (Pine Research) that has a built-in working and counter electrodes. The same pseudo refence electrode as colloidal CVs was placed in the upper well of the cuvette and referenced against $Fc^{+/0}$ prior to SEC experiments. Controlled potential electrolysis (CPE) were perform using the same potentiostat setup described above and spectral measurements were acquired with an Ocean Optics 20 W Tungsten Halogen white light source delivered to and from the sample through fiber-optic cables plumbed into the glovebox by a KF passthrough port. Spectra were collected on an OceanFX UV-visible detector against blank electrolyte baseline.

EPR spectroscopy.

All samples were prepared in 3:1 THF/toluene solvent mixtures with 30 uM of Si-vpy and corresponding [Co] equiv. Continuous wave EPR data were collected at 0.1 mW power and 10 G modulation amplitude using an X-band Bruker Elexsys E-500 spectrometer cooled to 50 K.

Surface-functionalized Si NCs purification procedure.

Purification following all Si NC surface functionalization reactions was conducted by precipitating the resulting products with acetonitrile (MeCN) in a 50-mL centrifuge tube followed by centrifugation at 10,000×*g* for 10 min. The supernatant was then decanted inside the glovebox and the solid was resuspended in 2 mL of toluene followed by repeating the precipitation and centrifugation steps for another 2-3 times. The Si NCs were then stored as colloids in either toluene or 3:1 THF/toluene mixture.

Si-fpy two-step synthesis.

1. Step 1: A mixture containing ca. 10–20 mg of 3.9 nm Si NCs, a catalytic amount of ABCN (~ 1 mg), and 1–1.5 mL of 1-dodecene was heated at 140 °C for ca. 10 min. The reaction mixture was then cooled to room temperature and 2 mL toluene was added followed by filtration through a 0.7 µm microfiber glass plug into a 50 mL centrifuge tube. The undersaturated Si-C_{12} NCs were then precipitated with MeCN and purified as described above.

2. Step 2: A toluene colloid (~1 mL) of the Si-C_{12} NCs undersaturated with dodecyl ligands from step 1 was added a catalytic amount of ABCN (~1 mg) and 250 μL of 4-formylpyridine. The reaction mixture was heated to 100 °C for 24 h and then cooled to ambient temperature. The resulting colloid was then filtered through a 0.7 μm microfiber glass plug into a 50 mL centrifuge tube followed by purification procedure described above.

Si-vpy one-pot synthesis.

A mixture containing ca. 10–20 mg of 3.9 nm Si NCs, a catalytic amount of ABCN (~1 mg), and 1–1.5 mL of 1-dodecene was heated to 140 °C in a vial equipped with a rubber septum. After the solution became translucent, ca. 5 min, 250 μL of 4-vinylpyridine was slowly added via micro-syringe in a dropwise fashion. The reaction mixture was heated at 140 °C for 12 h and then cooled to ambient temperature. The resulting mixture was filtered through a 0.7 μm microfiber glass plug into a 50 mL centrifuge tube and purified according to the procedure described above.

II. Supporting Data.

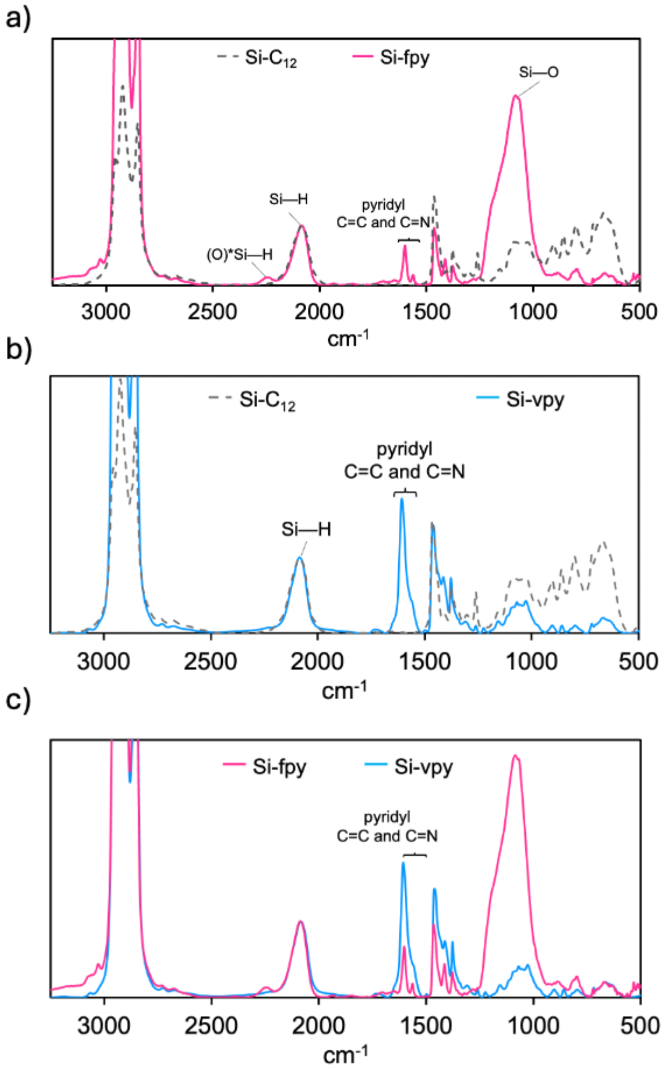

Figure S1. DRIFTS spectra overlay of (a) Si-fpy and Si-C_{12}, (b) Si-vpy and Si-C_{12}, and (c) Si-fpy and Si-vpy. All spectra normalized to the peak intensity of the $\nu_{Si\text{–}H}$ stretch at ~2090 cm^{-1}.

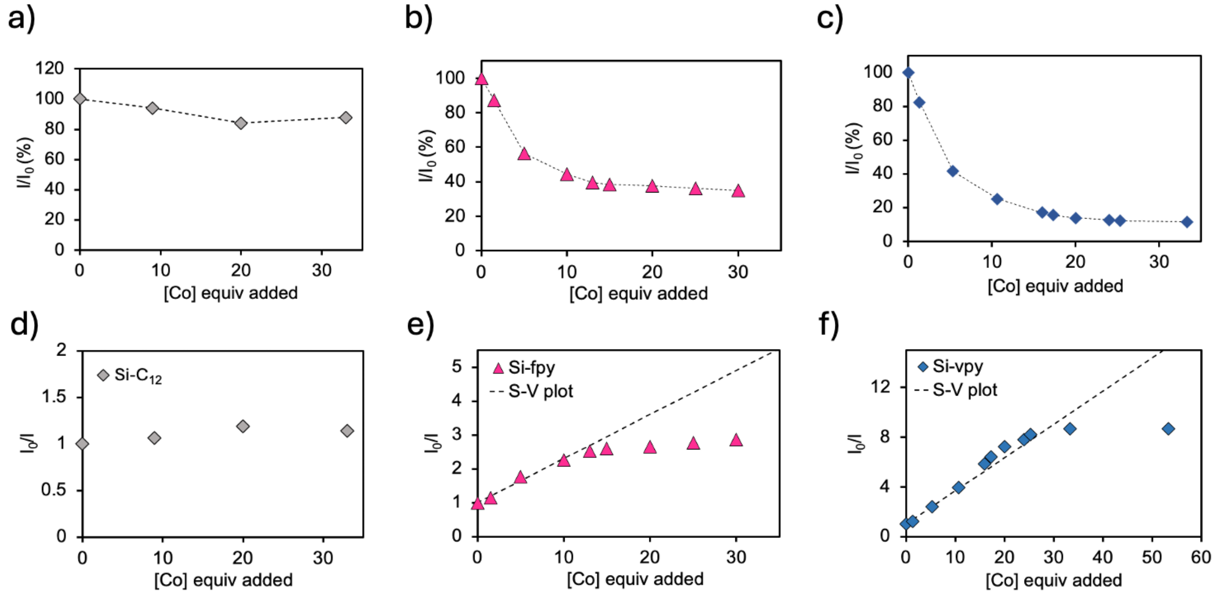

Figure S2. (a)–(c) PL quenching I/I_0 (%) vs. [Co] equiv added; and (d)–(f): PL I_0/I vs. [Co] equiv added of Si-C_{12} (gray–(a) and (d), Si-fpy (pink–(b) and (e), and Si-vpy (blue–(c) and (f). The dashed lines in panel (e) and (f) represent the expected PL I_0/I value from the Stern-Volmer (S-V) linear relationship for collisional PL quenching according to $I_0/I = 1 + K_{SV} \times [Q]$ (where K_{SV} = S-V constant and [Q] = quencher concentration). The S-V constants K_{SV} in each case are estimated from linear fitting of the data points at low [Co] equiv.

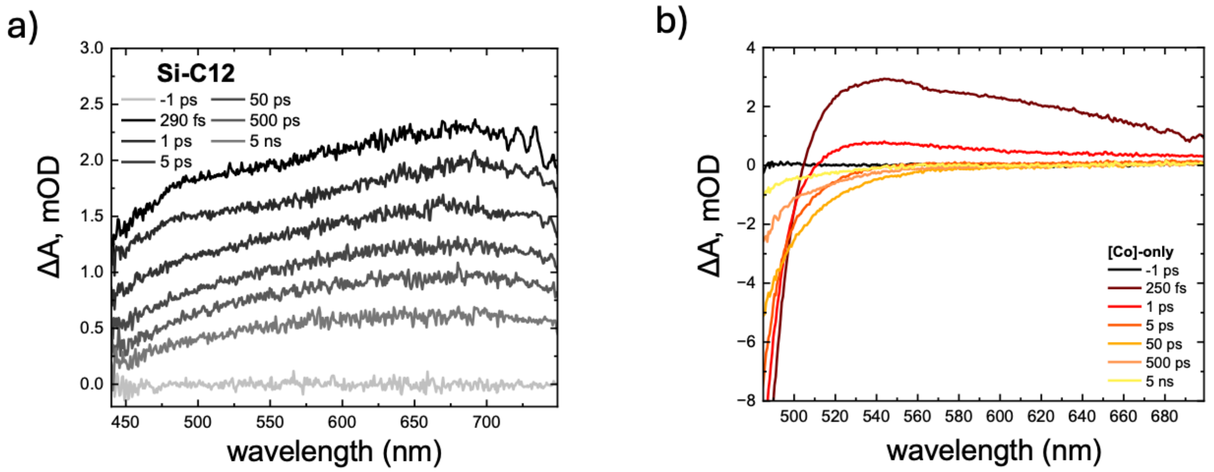

Figure S3. TAS data of (a) Si-C_{12} alone and (b) [Co] complex alone. Both were collected in 3:1 THF/toluene (v/v) solvent mixtures with 400 nm photoexcitation.

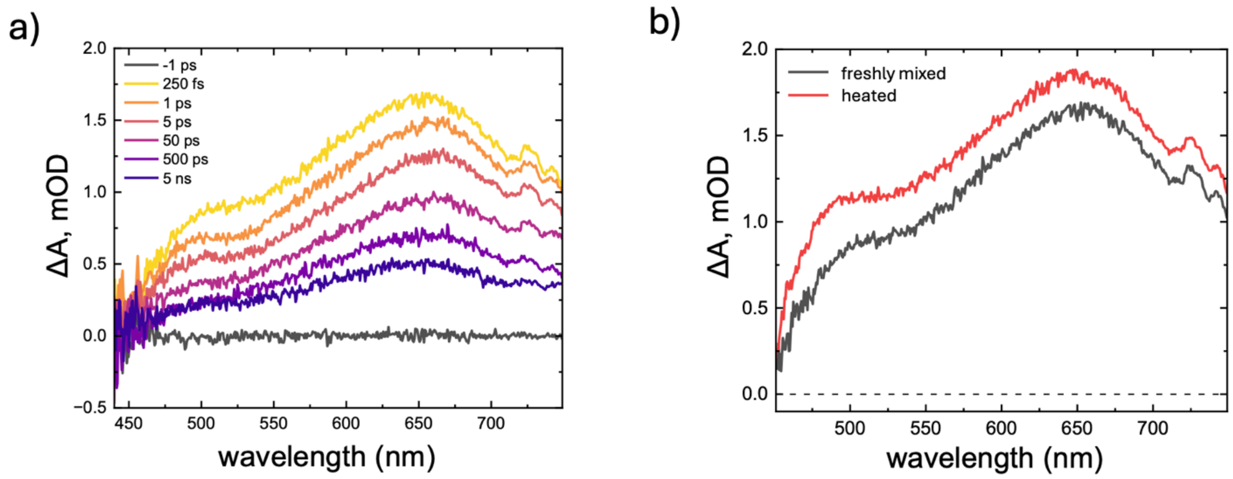

Figure S4. (a) TAS data of Si-vpy-[Co] saturated (~20 equiv [Co]) freshly mixed; (b) Overlay of TAS data at 250 fs of Si-vpy-[Co] freshly mixed and heated (50 °C, 12 h). These data do not prove that every surface-tethered [Co] complex is active in the hot electron injection events characterized by the 640 nm feature, but they instead show that a single Si-$vpy_{proximal}$-[Co] binding motif is all that is required to generate strong electronic coupling. And the slight increase in intensity after heating suggests that many [Co] units bind to the distal sites and got transferred to the proximal after heating.

CVs of Si-C_{12}, Si-vpy, and [Co] with added amounts of vpy. The cyclic voltammograms (CVs) of colloidal Si-C_{12} and Si-vpy are both collected in THF/toluene 3:1 (v/v) (Figure S5), and both do not display any reduction features until very negative potentials are applied. The CV of Si-C_{12} exhibits two cathodic peaks at potentials of E_{pc1} = –2.4 V and E_{pc2} = –2.9 V vs. $Fc^{+/0}$ and no corresponding oxidation wave, suggesting that Si-C_{12} reduction is irreversible. In contrast, the Si-vpy CV displays two broad reduction events at E_{pc1} = –2.65 V

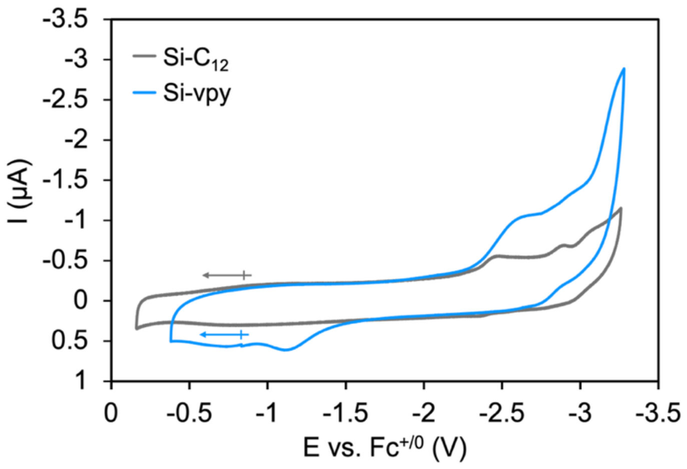

Figure S5. Overlaid CVs of Si-C_{12} (gray) and Si-vpy (light blue). Condition: 50 mM $TBAPF_6$ in 3:1 THF/toluene, scan rate: 50 mV/s.

and E_{pc2} = –2.85 V vs. $Fc^{+/0}$, albeit with higher cathodic currents as expected from incorporation of vpy into the insulating aliphatic C_{12} ligand shell (Figure S5). In addition, a re-oxidation wave with anodic peak potential of E_{pa} = –1.14 V is found for Si-vpy that is absent in Si-C_{12}, consistent with oxidation of reduced pyridyl groups and showing that the increased cathodic current found in the Si-vpy CV is likely a convolution of more facile reduction of the Si itself with reduction of surface-bound vpy.

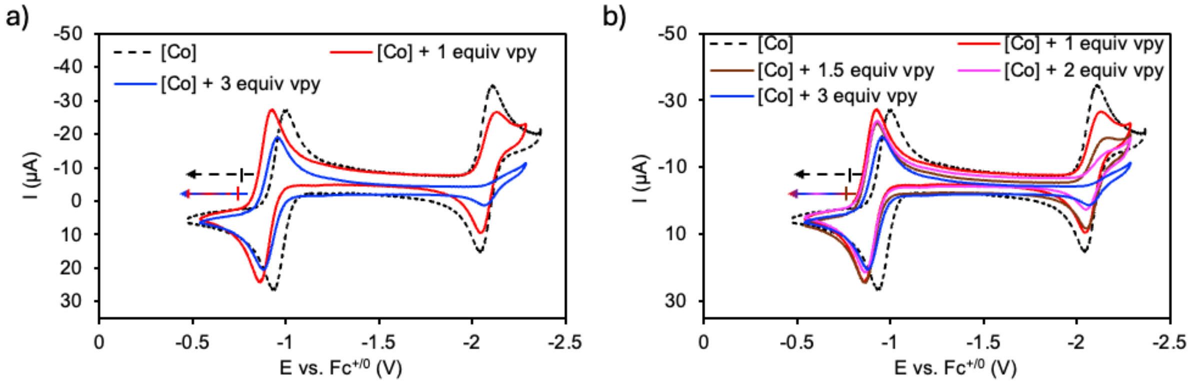

Figure S6. (a) Overlaid CVs of free [Co] (dashed black), [Co] + 1 equiv vpy (red) and [Co] + 3 equiv vpy (blue). (b) Plot (a) with additions of [Co] + 1.5 equiv vpy (brown) and [Co] + 2 equiv vpy (pink). CVs taken in MeCN solution containing 100 mM $TBAPF_6$, scan rate 100 mV/s.

EPR analysis

All EPR spectra of Si-vpy-[Co] contain two components: a major rhombic signal and a minor isotropic signal at g = 2.006. The minor component, which is also present in the EPR spectrum of Si-vpy, Figure S7a-b, is typical of the dangling bonds on Si NCs surface that we have observed previously and is characteristic of a surface radical from minor oxidization of the Si NC surface that arises from the surface functionalization chemistry.[6] The dominant signal is associated with Co^{II} centers having S = ½, which displays clear hyperfine structure coming from interacting with the Co^{II} I = 7/2 nuclear spin in the free [Co] sample (panel c). On the other hand, the Co^{II} signals in Si-vpy-[Co] display significant line broadening, especially below [Co] saturation points (panel d-f), which is attributed to unresolved weak hyperfine interaction with both ^{15}N on the coordinated pyridyl groups and surface ^{29}Si nuclei. Hyperfine interaction with ^{29}Si can also be seen in the zoomed in EPR spectrum of Si-vpy (panel b). At 20 equiv [Co] (panel g), the cobalt EPR signal of Si-vpy-[Co] is more defined, albeit still broadened compared to free [Co], allowing clear comparison between the freshly mixed (panel g) and heated samples (panel h). Consistent with electrochemical measurement, the two EPR spectra at 20 equiv display several differences in which the most apparent change is the significant drop in the signal intensity

of the dangling bonds accompanied with a slight decrease in [Co] signal intensity (overlaid spectra is in the main text Figure 4c). The decrease of both signals indicates that there are several [Co] sites that have their spins antiferromagnetically coupled with the dangling bonds on the surface. Additionally, further broadening of [Co] hyperfine structure at ca. g ~2.21 (green ovals) occurs upon heating Si-vpy-[Co] with 20 equiv [Co] indicating additional hyperfine interaction with ^{29}Si nuclei. These observations provide strong support that heating results in primarily proximal [Co] binding and enables the strong electronic communication between the Si NC and [Co].

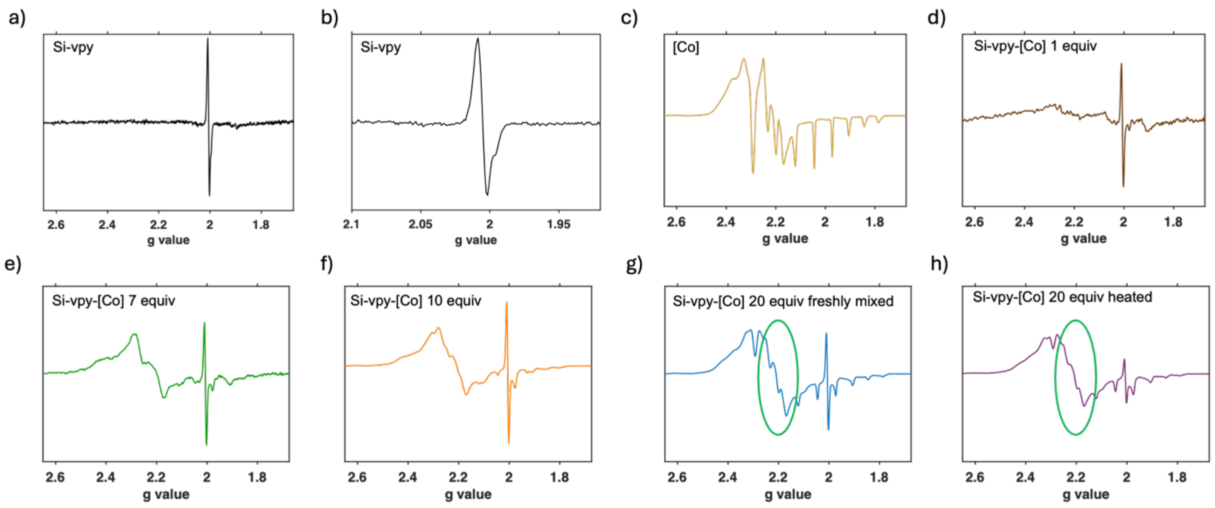

Figure S7. X-band EPR spectra of (a) Si-vpy, (b) zoomed in Si-vpy, (c) [Co], (d)-(f) Si-vpy-[Co] at 1, 7, and 10 equiv [Co], and (g)-(h) Si-vpy-[Co] at 20 equiv [Co] freshly mixed and heated. All samples were in a mixture of THF/tol and all EPR measurements were taken at 50 K.

CV analyses for [Co] binding environment in Si-vpy-[Co] and Si-fpy-[Co]: The binding environment of [Co] to Si-fpy should result in exclusively mono-axial coordination due to the absence of the distal sites. Indeed, the CV of Si-fpy-[Co] at 1 equiv [Co] maintains both [Co] redox waves and exhibits a slightly suppressed $[Co]^{-/2-}$ redox wave above –2.0 V vs. $Fc^{+/0}$ (Figure S8a), similar to behavior observed in the control experiment where 1 to 1.5 equiv free vpy is added to free [Co] (Figure S6a,b). Both [Co] redox waves in Si-fpy-[Co] (1 equiv [Co]) are maintained after heating (Figure S8b) indicating that there is no change in [Co] environment upon heating and mono-axial (and likely mono-axial proximal) is the exclusive binding mode in Si-fpy-[Co].

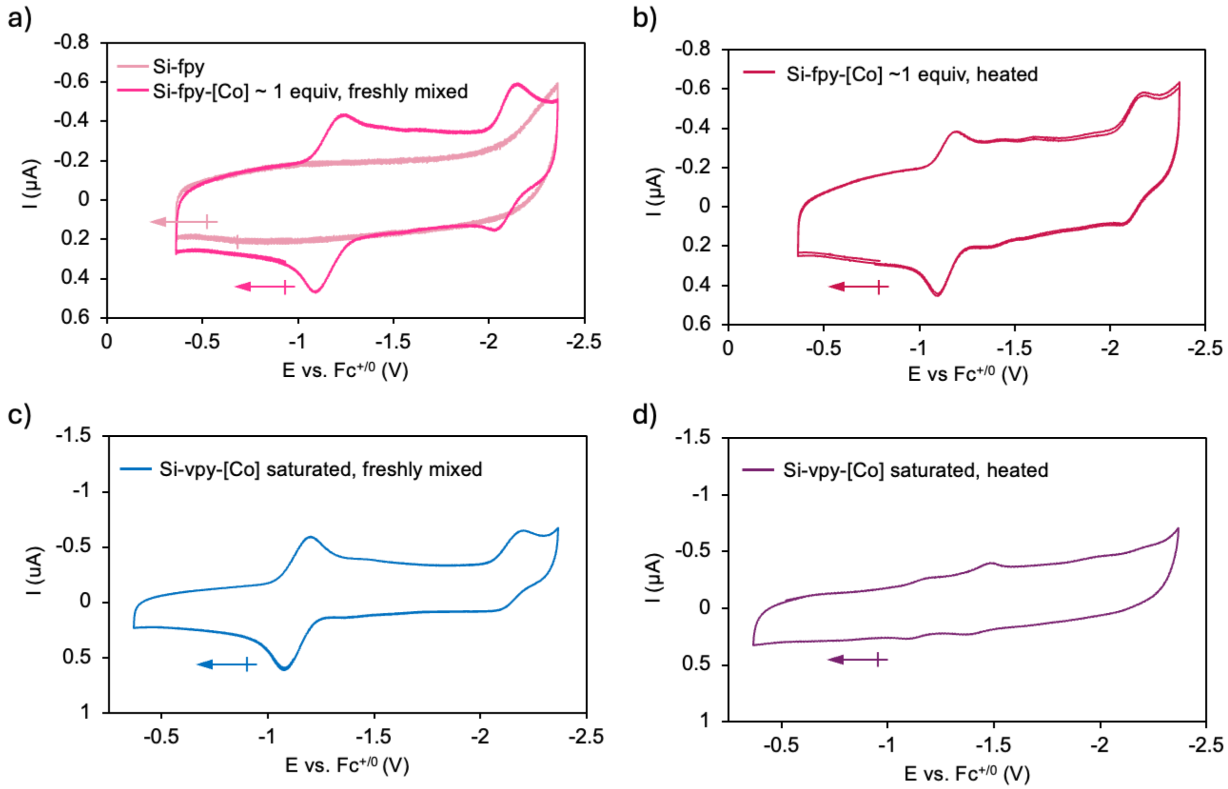

Figure S8. (a) Overlaid CVs of Si-fpy (magenta) and Si-fpy-[Co] (light magenta) at 1 equiv [Co]. (b) CV of Si-fpy-[Co] at 1 equiv [Co] heated at 50 °C, 12 h. (c) CV of Si-vpy-[Co] at 20 equiv [Co] freshly mixed. (d) CV of Si-vpy-[Co] at 20 equiv [Co] heated at 50 °C, 12 h. Condition: CVs taken in 3:1 THF/tol containing 50 mM $TBAPF_6$, scan rate 50 mV/s.

Though the disappearance of the second redox wave at ca. –2.1 V in a freshly mixed Si-vpy-[Co] sample with ~1 equiv [Co] shows that bis-axial pyridyl binding occurs immediately (Figure 4a, middle panel), it does not reveal whether binding occurs through the distal-distal or proximal-distal motifs. Additional insight is provided from the CV of Si-vpy-[Co] with 20 equiv [Co] (Figure S8c,d). As expected for this sample with ~1:1 [Co]/pyridyl, the CV for Si-vpy-[Co] (20 equiv [Co]) resembles that of free [Co] mixed with 1 to 1.5 equiv vpy in which the second redox wave associated with the $[Co]^{-/2-}$ couple is observed but is slightly suppressed (Figure S6a,b). Also, like Si-vpy-[Co] (1 equiv) CV, heating this Si-vpy-[Co] (20 equiv [Co]) sample largely suppresses both [Co] redox features (Figure S8d). These experiments imply that initial [Co] binding to Si-vpy occurs via a mixture of distal and proximal motifs (either mono- or bis-axial depending on [Co] concentration) and that heating drives proximal coordination as the dominant binding mode, Figure S9. Whether the proximal coordination is mono- or bis-axial does not impact the CV results at low [Co] concentration due to the charge screening possible via strong electronic coupling in Si-vpy-[Co] as discussed in the main text. At high [Co]

concentrations, residual redox features are found suggesting a minor contribution from distal binding motifs as shown in Figure S9b.

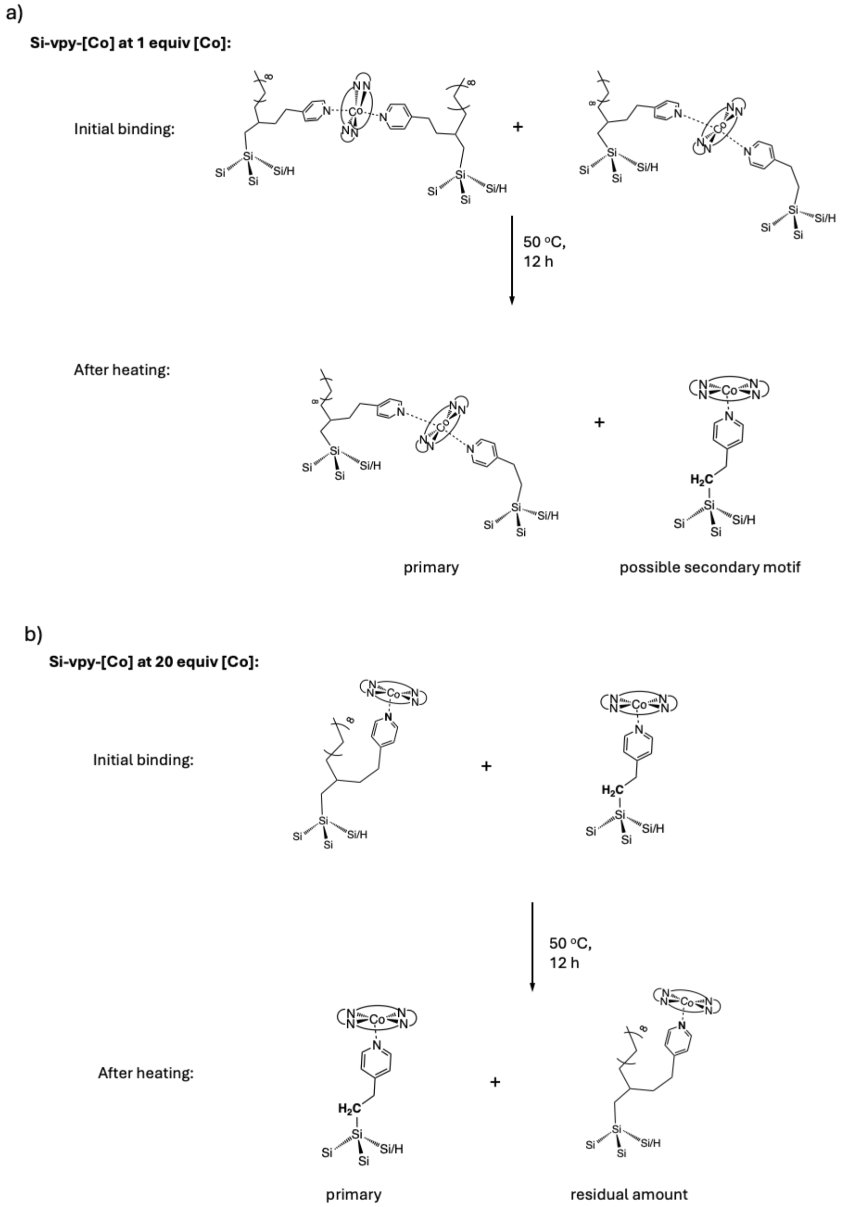

Figure S9. Illustration of the hypothesized change in binding motif from freshly mixed to heating (50 °C, 12 h) Si-vpy-[Co] at (a) 1 equiv [Co] and (b) at 20 equiv [Co].

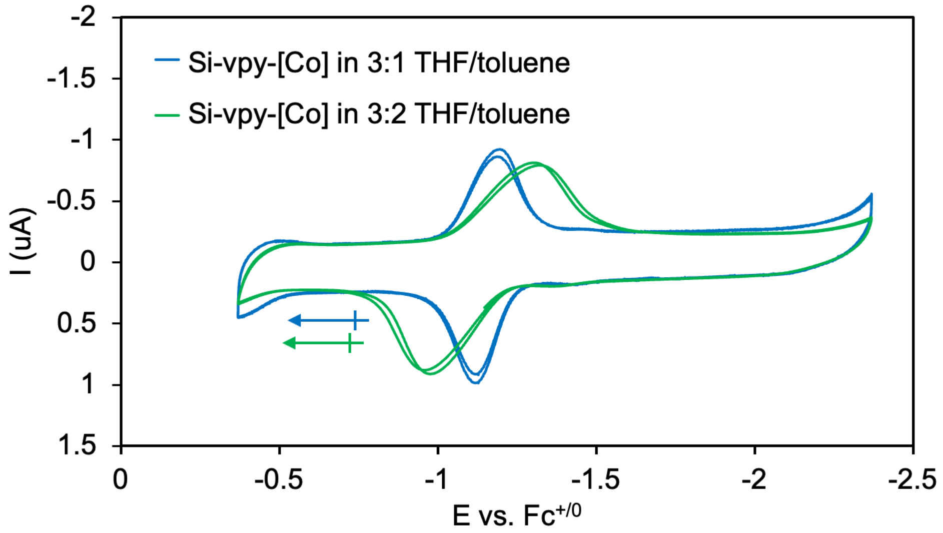

Figure S10. CVs of Si-vpy-[Co] at 1 equiv [Co] in 3:1 (blue) and 3:2 (green) THF/tol ratio. Condition: 50 mM $TBAPF_6$ supporting electrolyte for 3:1 THF/toluene; slightly < 50 mM $TBAPF_6$ in 3:2 THF/toluene due to lower solubility of $TBAPF_6$ in the less polar 3:2 THF/toluene solvent; scan rate 50 mV/s. The first redox wave in the Si-vpy-[Co] CV at 1 equiv [Co] has a narrow peak-to-peak separation that along with its sharpness might suggest that there is adsorption of [Co] to the GCE instead of binding to Si-vpy. However, this control experiment disproves this idea. As the THF/toluene solvent ratio is slightly decreased (less polar electrolyte), the wave shape becomes more diffusional with larger redox peak separation, consistent with Si-vpy-[Co] diffusing more easily in an electrolyte with more toluene as would be expected for these primarily aliphatic-terminated Si NCs.

Analysis of CVs in the high potential region: A qualitative assessment of the barriers to electrochemical charge transfer is derived from scanning the CV towards more cathodic potentials where additional reduction features emerge. CVs are collected with Si-vpy and Si-vpy-[Co] with 1 equiv [Co] freshly mixed and heated (Figure S11). The cathodic current for Si-vpy-[Co] rises steadily at low potentials below –2.0 V that overlays closely with that of Si-vpy (Figure S9a). At higher cathodic potentials above –2.0 V, dramatic changes occur. The onset potential for the sharp current rise at ca. –2.3 V is shifted positively by 174 mV in the CV of heated Si-vpy-[Co] compared with that of Si-vpy and by 111 mV relative to freshly mixed Si-vpy-[Co] (Figure S9b). The shift in onset potential for the sharp current rise is consistent with [Co] binding simply lowering the barrier to charge transfer—as would be expected for inserting a redox-active molecule within a primarily hydrophobic molecular interfacial layer at the Si-vpy NC surface. But given other changes to the CV data, we offer an alternative hypothesis. Though undoubtedly some of the onset potential shift can be ascribed to a lower barrier height, the observation that [Co]-localized redox features above

–2.5 V in Si-vpy-[Co] freshly mixed (light blue) are no longer resolved after heating (purple) and the CV shape following heating closely matches that of Si-vpy with no [Co] (dashed blue) suggests that electrochemical charge transfer occurs directly into high energy hybridized states.

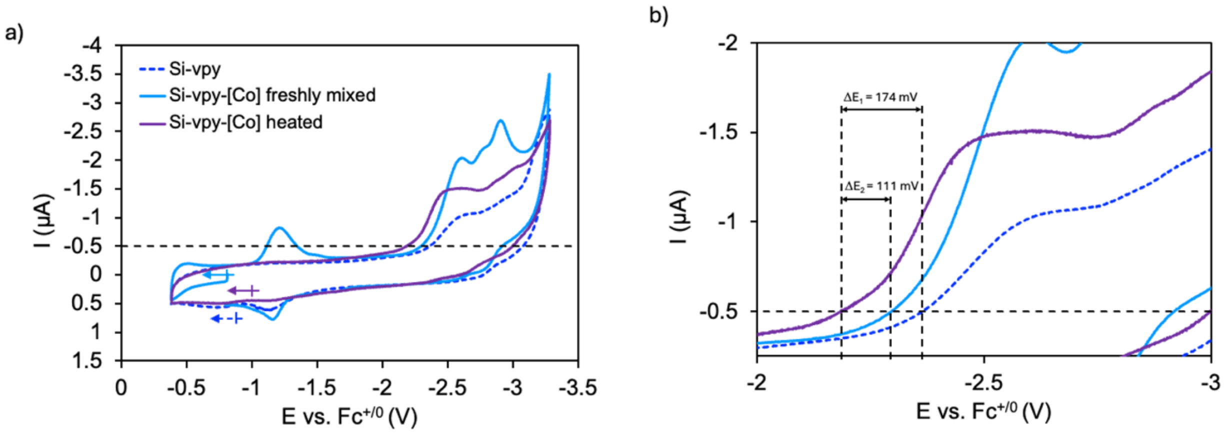

Figure S11. (a) Overlaid CVs of Si-vpy (dashed blue), Si-vpy-[Co] freshly mixed (light blue) and Si-vpy-[Co] heated (purple) at 1 equiv [Co]. (b) Zoomed in CVs highlighting the positive shift of onset potentials for the sharp current rise region near –2.3 V vs. $Fc^{+/0}$.

SEC analysis. Additional support for the energetic hybridization within Si-vpy-[Co] is provided by SEC measurements on the heated Si-vpy-[Co] sample at 1 equiv [Co], Figure S12. We plot the potentials where SEC chronoamperometry experiments are conducted overlaid on the free [Co] CV in Figure 12a, the current vs. time (I-t) plot of the chronoamperometry prior to collecting SEC absorption measurements in Figure S12b, and the raw absorbance and difference spectra at various potentials in Figure S12c-d (Note: panels c-d are reproduced from main text Figure 4d-e for easy reference). Consistent with CV measurements, the I-t data reveal that there is negligible charged passed at –1.3 V vs. $Fc^{+/0}$ and the absorbance spectrum is indistinguishable from that of Si-vpy NCs (cf. Figure 2f). At potentials close to the sharp current rise onset in CV (–2.0 V and –2.3 V), charge is passed to an electrochemically active species with the I-t profiles following the exponential current decay described by the Cottrell equation typically seen for diffusional-controlled processes represented by the dashed black line in Figure S12b. However, no characteristic absorption from reduced [Co] species is found in the absorbance spectra collected at these two potentials (Figure S12c,d, light blue and dark green traces for SEC of Si-vpy-[Co] at –2.0 V and –2.3 V, respectively).

Dramatically different SEC response occurs at –2.5 V, where the Si-vpy-[Co] I-t deviates significantly from the Cottrell equation (Figure S12b, purple trace), suggesting multiple charge equivalents are transferred as a single redox species reaches the electrode. In the corresponding absorbance spectrum, two new broad, strong absorption features emerge at 502 and 535 nm along with a weaker absorption peak at 614 nm after chronoamperometry at –2.5 V (Figure S12c,d, purple trace). These new features resemble but are distinct from the energies of absorption features observed in the SEC of free [Co] held at –2.55 V (489, 534, 623 nm) corresponding to irreversible reduction events beyond the 2nd [Co] reversible redox wave (i.e., 3rd or higher order reductions, Figure S12e, green trace).

The observation that discrete absorption features related to [Co]-based reduction are only present at extreme cathodic potentials and absent at –2.0 to –2.3 V suggests that at these lower but still highly reducing potentials—well above the reversible [Co] redox potentials—the charge electrochemically transferred into Si-vpy-[Co] is not electrochemically localized on [Co] but rather is delocalized within new electronic states within Si-vpy-[Co].

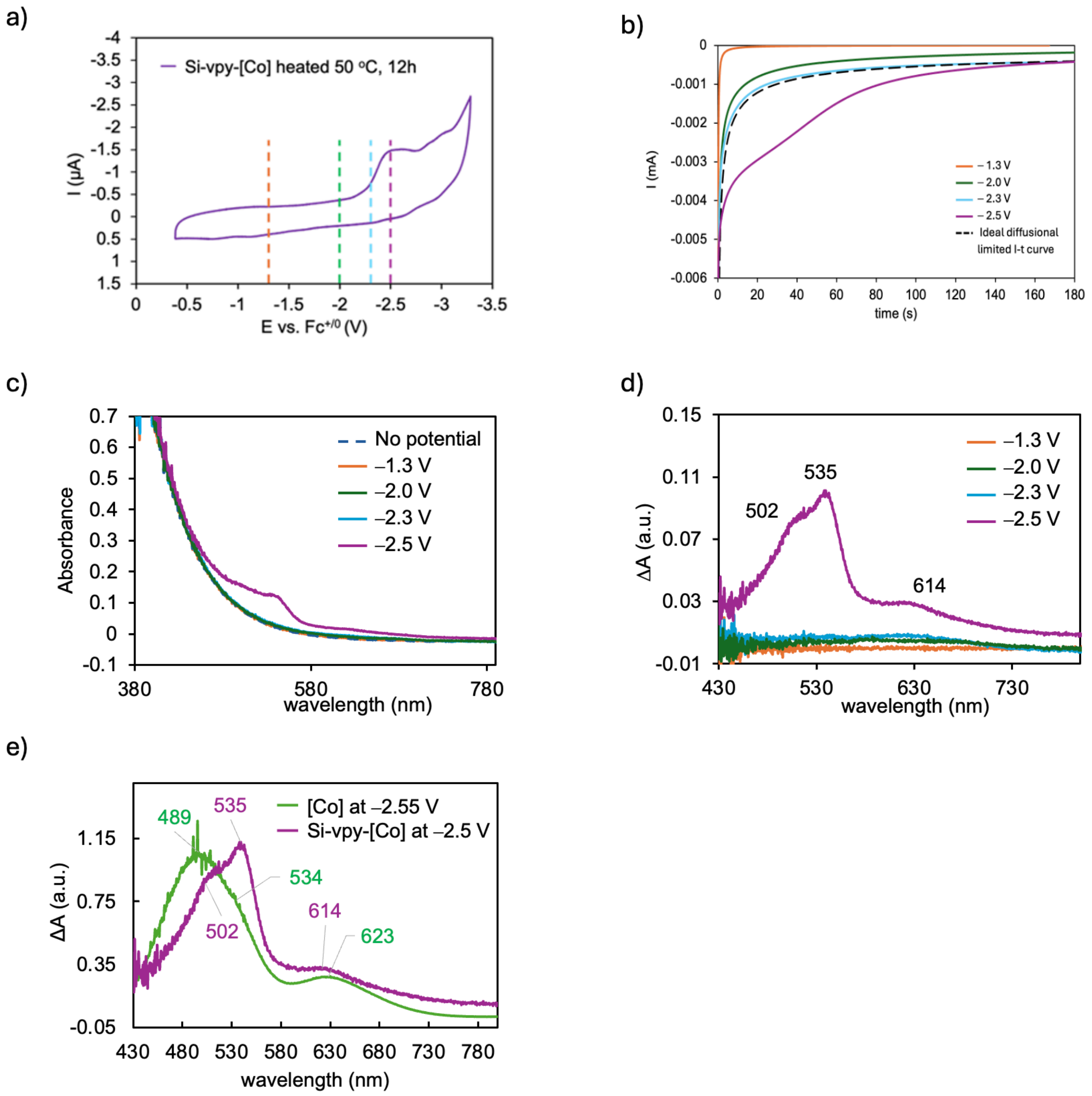

Figure S12. (a) CV of Si-vpy-[Co] at 1 equiv [Co] heated with dashed lines indicating potentials held for SEC measurements. (b) I-t curve from the chronoamperometry during SEC measurement of Si-vpy-[Co]. (c) Raw SEC spectra of Si-vpy-[Co] heated. (d) Difference SEC spectra from (b) by subtracting absorbance from Si-vpy-[Co] spectrum at no applied potential. (e) Difference SEC spectrum of Si-vpy-[Co] at –2.5 V (purple) overlaid with difference SEC spectrum of free [Co] at –2.55 V (green).

III. Computational Details

Structure generation. Initial Si NC geometries were created from the conventional diamond-cubic unit cell of silicon (space group Fd-3m, a = 5.47 Å) using Pymatgen.[7] A cubic supercell of a sufficient size to contain the desired Si NC was created with CubicSupercellTransformation. The atom closest to the cube center was identified, and all atoms within a spherical cutoff equal to half the target diameter were retained to form a centered Si NC. To eliminate surface dangling bonds and better mimic realistic Si NC surfaces, hydrogen passivation was applied using the Atomic Simulation Environment (ASE) package and neighbor list functionality to determine local bonding environment.[8] Each Si atom's coordination was analyzed, and hydrogen atoms are placed to complete tetrahedral coordination using a Si–H bond length of 1.497 Å (i.e., Si atoms with 3, 2, or 1 Si neighbors receive 1, 2, or 3 H atoms, respectively, oriented to preserve local tetrahedral geometry.)

Calculations. All density functional theory (DFT) calculations were performed using ORCA (version 5.0.3).[9,10] The M06-L meta-GGA functional was employed together with the def2-TZVP valence triple-zeta basis set for all atoms.[11,12] Solvent effects were included using the CPCM implicit solvation model with acetonitrile as the dielectric medium.[13] The RIJCOSX approximation and def2/J auxiliary basis set are used to accelerate the Coulomb and exchange integrals, in combination with a finer numerical integration grid (defgrid3).[14] Self-consistent field (SCF) convergence is enforced using the DIIS algorithm with TightSCF criteria.[15] To further improve convergence stability, the CNVZerner damping scheme is applied with specific dampening parameters (damp factor: 0.9, erroff: 0.05, minimum: 0.1, maximum: 0.99).[16] All atoms in the system are allowed to relax.

Mulliken population analyses were extracted from the ORCA output and parsed into a structured dataset containing the molecular orbital (MO) index, energy, spin channel, orbital occupancy, and atom-resolved Mulliken contributions.[17] For each spin channel, the orbitals are first sorted by energy, and the HOMO is identified as the highest-energy orbital with an occupancy ≥ 1.0, while the LUMO is assigned as the first unoccupied orbital (occupancy ≈ 0.0) immediately above it. For each orbital analyzed, the Mulliken contributions were summed over all atomic basis functions belonging to each atomic species. Atomic centers are then grouped into chemically two meaningful categories: Si and [Co] plus linker (transition metal (Co) and heteroatoms C, N, O, B, F) to yield an element-resolved orbital composition expressed as a fraction of the total Mulliken population per orbital. This analysis allows us to quantify the orbital character as Si-centered, [Co]/molecular tether-centered, or hybridized. All structure and orbital visualizations were produced using VESTA software and are visualized at a 5E-3 a.u. isosurface level.[18]

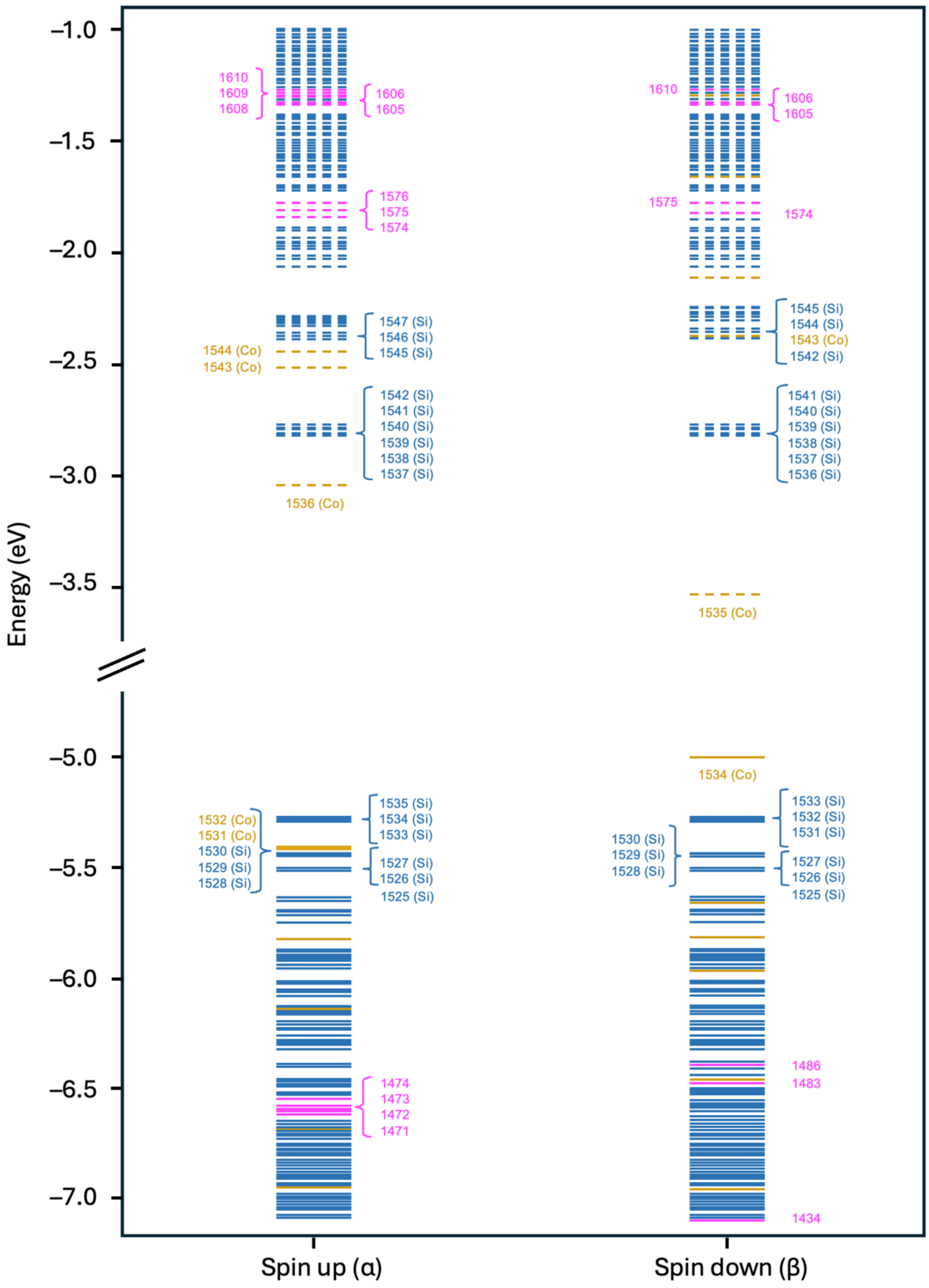

Figure S13. Energy diagram of Si-vpy-[Co] MOs from HOMO-100 to LUMO+100 with blue lines indicating Si-based orbitals (Si), yellow lines [Co]-based orbitals (Co), and magenta lines hybridized states between Si and [Co + linker]. Solid lines are occupied orbitals and dashed lines unoccupied orbitals.

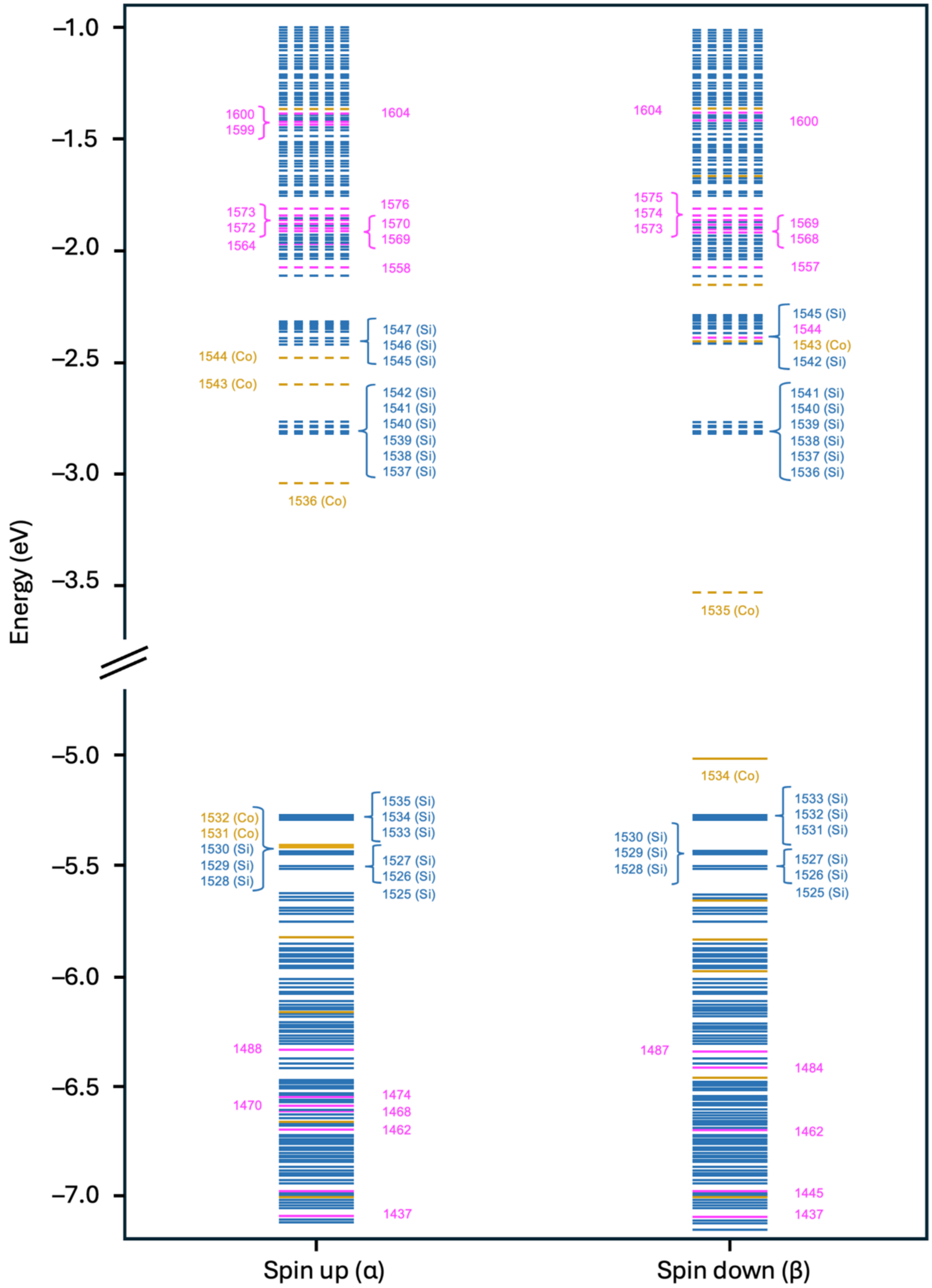

Figure S14. Energy diagram of Si-fpy-[Co] MOs from HOMO-100 to LUMO+100 with blue lines indicating Si-based orbitals (Si), yellow lines [Co + linker]-based orbitals (Co), and magenta lines hybridized states between Si and [Co + linker]. Solid lines are occupied orbitals and dashed lines unoccupied orbitals.

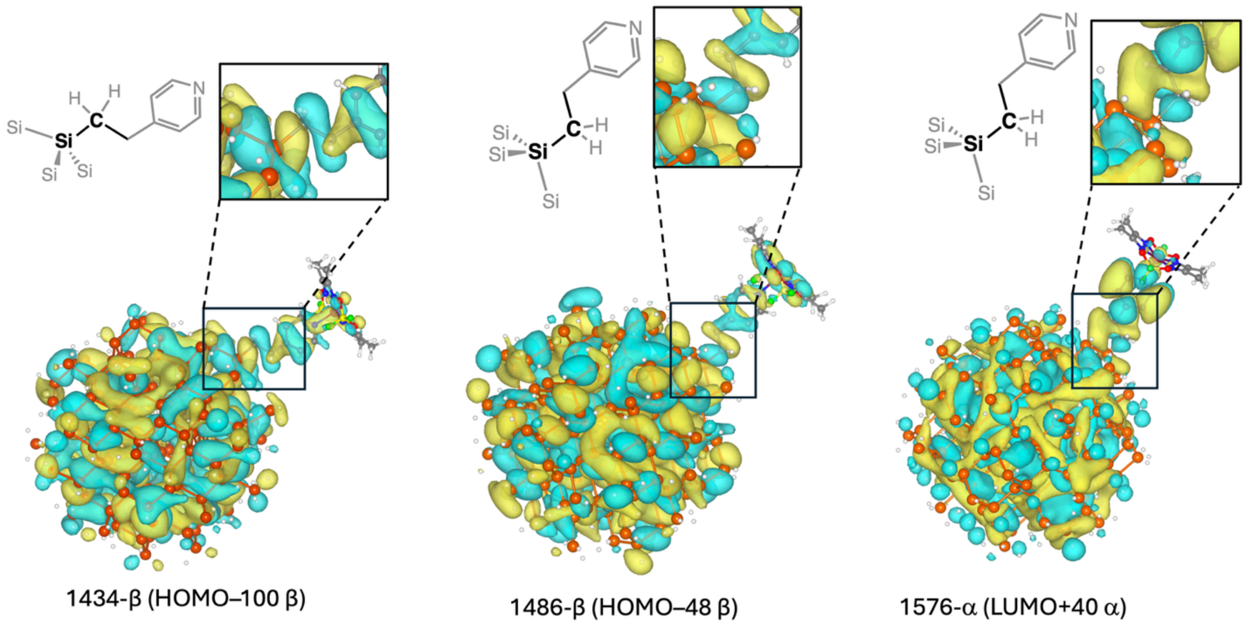

Figure S15. Selected Si-vpy-[Co] hybridized MOs that display *Si–C σ-bonding interactions (where *Si represents a surface Si atom).

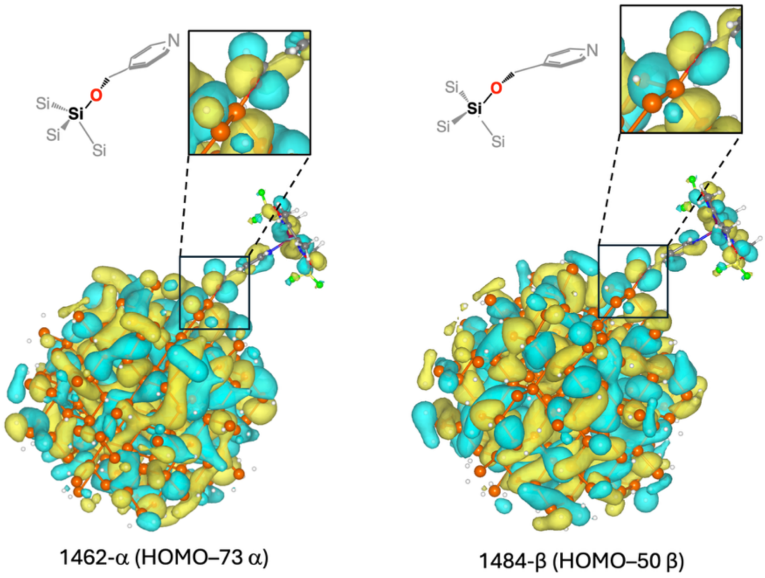

Figure S16. Selected Si-fpy-[Co] hybridized MOs that display *Si–O π-antibonding interactions (where *Si represents surface Si atoms).

References

(1) Connolly, P.; Espenson, J. H. Cobalt-Catalyzed Evolution of Molecular Hydrogen. *Inorg. Chem.* **1986**, *25*, 2684–2688. https://doi.org/10.1021/ic00236a006.

(2) *Catalytic, Sulfur-Free Chain Transfer Agents That Alter the Mechanical Properties of Cross-Linked Photopolymers | Journal of the American Chemical Society*. https://pubs.acs.org/doi/full/10.1021/jacs.3c03811 (accessed 2025-05-23).

(3) Wheeler, L. M.; Anderson, N. C.; Palomaki, P. K. B.; Blackburn, J. L.; Johnson, J. C.; Neale, N. R. Silyl Radical Abstraction in the Functionalization of Plasma-Synthesized Silicon Nanocrystals. *Chem. Mater.* **2015**, *27*, 6869–6878. https://doi.org/10.1021/acs.chemmater.5b03309.

(4) Carroll, G. M.; Limpens, R.; Neale, N. R. Tuning Confinement in Colloidal Silicon Nanocrystals with Saturated Surface Ligands. *Nano Lett.* **2018**, *18*, 3118–3124. https://doi.org/10.1021/acs.nanolett.8b00680.

(5) Saund, S. S.; Dabak-Wakankar, A.; Gish, M. K.; Neale, N. R. Silicon Nanocrystal Hybrid Photocatalysts as Models to Understand Solar Fuels Producing Assemblies. *Sustainable Energy Fuels* **2024**, *8*, 403–409. https://doi.org/10.1039/D3SE01512B.

(6) Limpens, R.; Pach, G. F.; Mulder, D. W.; Neale, N. R. Size-Dependent Asymmetric Auger Interactions in Plasma-Produced n- and p-Type-Doped Silicon Nanocrystals. *J. Phys. Chem. C* **2019**, *123*, 5782–5789. https://doi.org/10.1021/acs.jpcc.9b00223.

(7) Ong, S. P.; Richards, W. D.; Jain, A.; Hautier, G.; Kocher, M.; Cholia, S.; Gunter, D.; Chevrier, V. L.; Persson, K. A.; Ceder, G. Python Materials Genomics (Pymatgen): A Robust, Open-Source Python Library for Materials Analysis. *Computational Materials Science* **2013**, *68*, 314–319. https://doi.org/10.1016/j.commatsci.2012.10.028.

(8) Hjorth Larsen, A.; Jørgen Mortensen, J.; Blomqvist, J.; Castelli, I. E.; Christensen, R.; Dułak, M.; Friis, J.; Groves, M. N.; Hammer, B.; Hargus, C.; Hermes, E. D.; Jennings, P. C.; Bjerre Jensen, P.; Kermode, J.; Kitchin, J. R.; Leonhard Kolsbjerg, E.; Kubal, J.; Kaasbjerg, K.; Lysgaard, S.; Bergmann Maronsson, J.; Maxson, T.; Olsen, T.; Pastewka, L.; Peterson, A.; Rostgaard, C.; Schiøtz, J.; Schütt, O.; Strange, M.; Thygesen, K. S.; Vegge, T.; Vilhelmsen, L.; Walter, M.; Zeng, Z.; Jacobsen, K. W. The Atomic Simulation Environment—a Python Library for Working with Atoms. *J. Phys.: Condens. Matter* **2017**, *29*, 273002. https://doi.org/10.1088/1361-648X/aa680e.

(9) Neese, F. The ORCA Program System. *WIREs Computational Molecular Science* **2012**, *2*, 73–78. https://doi.org/10.1002/wcms.81.

(10) Neese, F. Software Update: The ORCA Program System—Version 5.0. *WIREs Computational Molecular Science* **2022**, *12*, e1606. https://doi.org/10.1002/wcms.1606.

(11) Zhao, Y.; Truhlar, D. G. A New Local Density Functional for Main-Group Thermochemistry, Transition Metal Bonding, Thermochemical Kinetics, and Noncovalent Interactions. *J. Chem. Phys.* **2006**, *125*. https://doi.org/10.1063/1.2370993.

(12) Weigend, F.; Ahlrichs, R. Balanced Basis Sets of Split Valence, Triple Zeta Valence and Quadruple Zeta Valence Quality for H to Rn: Design and Assessment of Accuracy. *Phys. Chem. Chem. Phys.* **2005**, *7*, 3297–3305. https://doi.org/10.1039/B508541A.

(13) Barone, V.; Cossi, M. Quantum Calculation of Molecular Energies and Energy Gradients in Solution by a Conductor Solvent Model. *J. Phys. Chem. A* **1998**, *102*, 1995–2001. https://doi.org/10.1021/jp9716997.
(14) Neese, F.; Wennmohs, F.; Hansen, A.; Becker, U. Efficient, Approximate and Parallel Hartree–Fock and Hybrid DFT Calculations. A ‘Chain-of-Spheres’ Algorithm for the Hartree–Fock Exchange. *Chemical Physics* **2009**, *356*, 98–109. https://doi.org/10.1016/j.chemphys.2008.10.036.
(15) Pulay, P. Convergence Acceleration of Iterative Sequences. the Case of Scf Iteration. *Chemical Physics Letters* **1980**, *73*, 393–398. https://doi.org/10.1016/0009-2614(80)80396-4.
(16) Zerner, M. C.; Hehenberger, M. A Dynamical Damping Scheme for Converging Molecular Scf Calculations. *Chemical Physics Letters* **1979**, *62*, 550–554. https://doi.org/10.1016/0009-2614(79)80761-7.
(17) Mulliken, R. S. Electronic Population Analysis on LCAO–MO Molecular Wave Functions. I. *J. Chem. Phys.* **1955**, *23*, 1833–1840. https://doi.org/10.1063/1.1740588.
(18) Momma, K.; Izumi, F. VESTA 3 for Three-Dimensional Visualization of Crystal, Volumetric and Morphology Data. *J Appl Cryst* **2011**, *44*, 1272–1276. https://doi.org/10.1107/S0021889811038970.
